# Supplementary figures and images for: The effect of health behavior interventions to manage Type 2 diabetes on the quality of life in low-and middle-income countries: A systematic review and meta-analysis
Source: PLoS One. 2023 Oct 16;18(10):e0293028. doi: 10.1371/journal.pone.0293028 (PMC10578590; doi:10.1371/journal.pone.0293028)

**S1 Fig. R codes used in the meta-analysis**


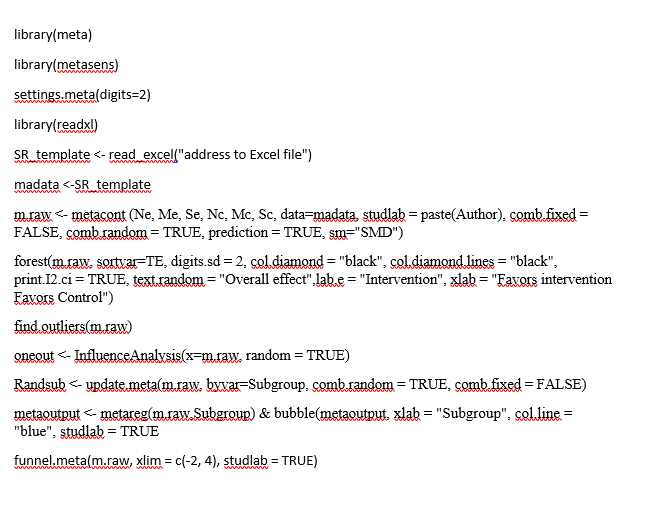

Supplement: S1 Fig — (DOCX) [file pone.0293028.s002.docx]

**S2 Fig. Funnel plot of Mean QOL**

***
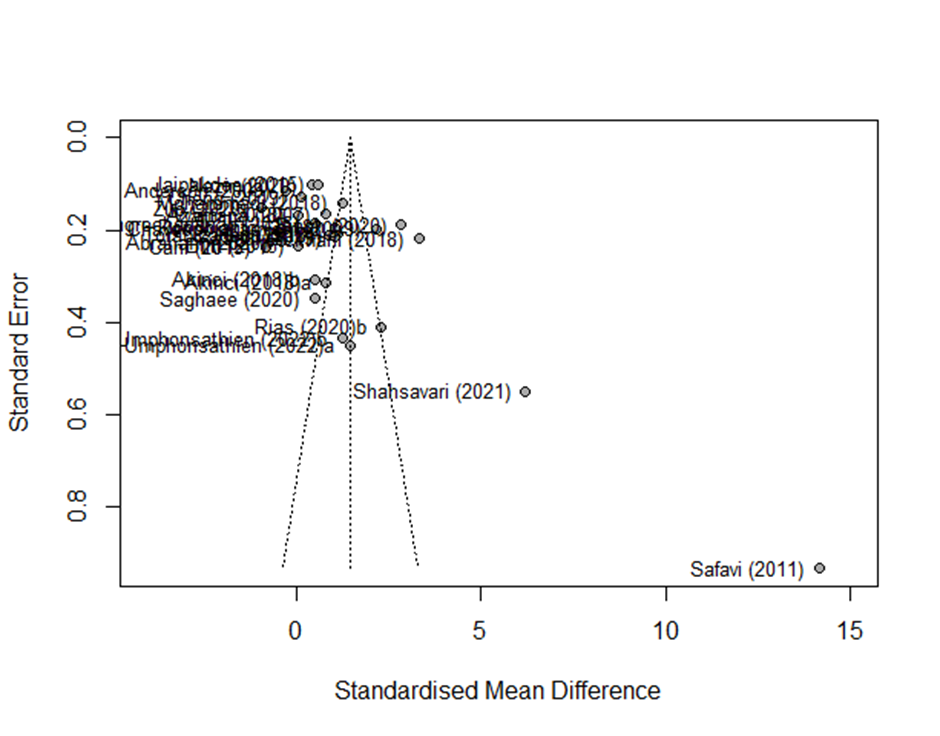
***

Supplement: S2 Fig — (DOCX) [file pone.0293028.s003.docx]

**S3 Fig. Funnel plot of Mean Physical Component Summary**

***
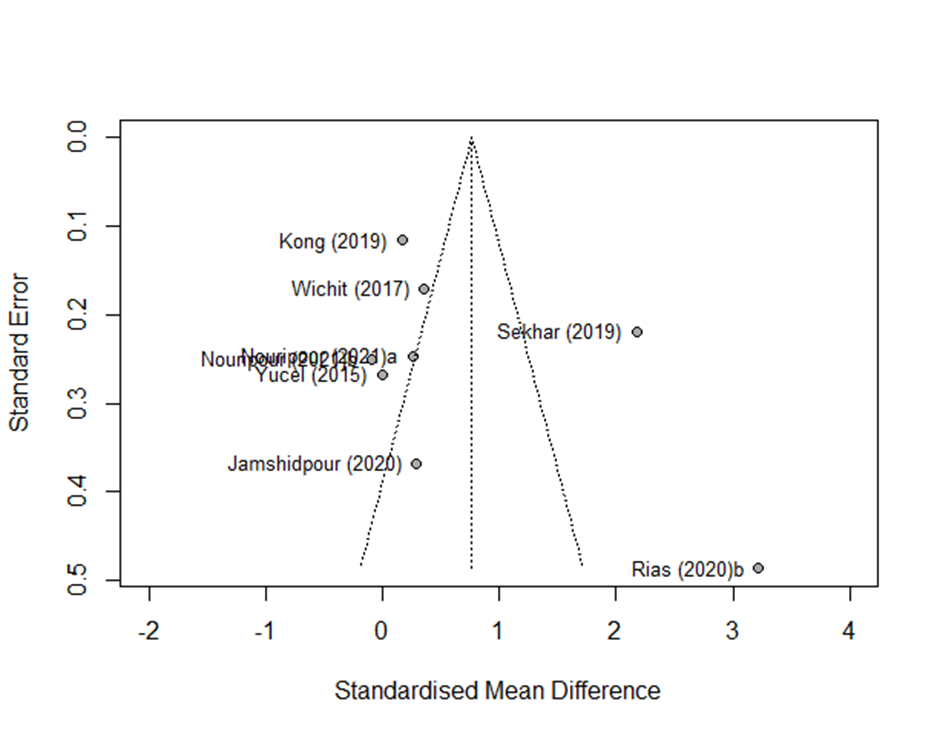
***

Supplement: S3 Fig — (DOCX) [file pone.0293028.s004.docx]

**S4 Fig. Funnel plot of Mean Mental Component Summary***
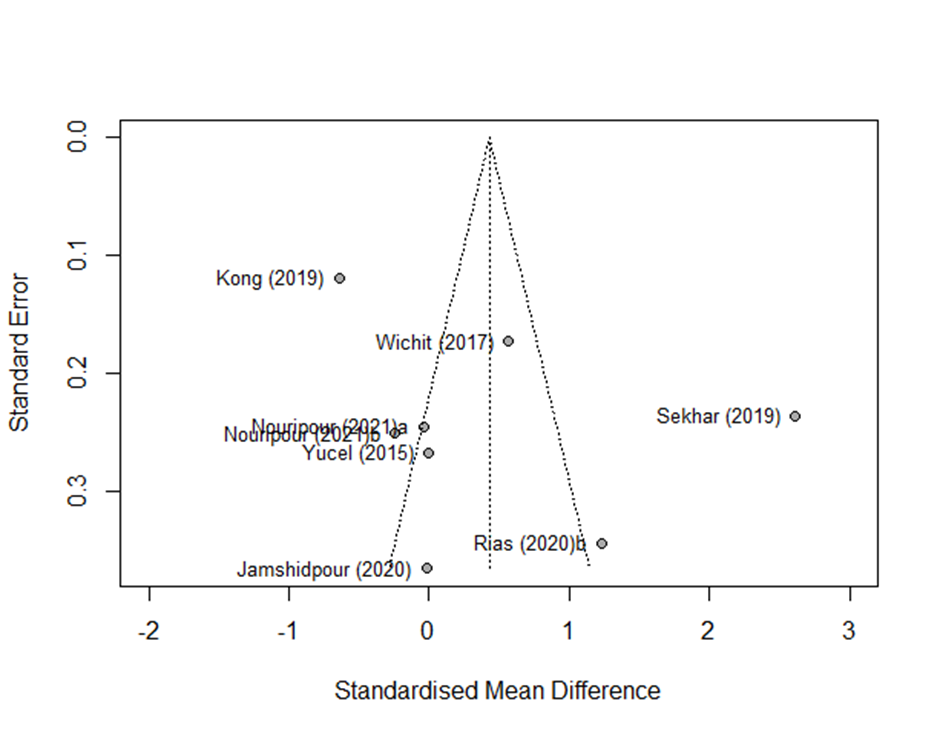
*

Supplement: S4 Fig — (DOCX) [file pone.0293028.s005.docx]
