# Supplementary material for: The effect of health behavior interventions to manage Type 2 diabetes on the quality of life in low-and middle-income countries: A systematic review and meta-analysis
Source: PLoS One. 2023 Oct 16;18(10):e0293028. doi: 10.1371/journal.pone.0293028 (PMC10578590; doi:10.1371/journal.pone.0293028)
Supplement: S1 Table — (DOCX) [file pone.0293028.s006.docx]

|  | **P**opulation | BOs | **I**ntervention | BOs | **O**utcome | BOs | **S**tudy design |
| --- | --- | --- | --- | --- | --- | --- | --- |
| Key concept | Type-2 Diabetes | AND | Health behaviour intervention | AND | Quality of life | AND | RCT |
| Controlled vocabulary terms | “Diabetes Mellitus, Type 2”[MeSH] |  | "health behaviour"[MeSH] |  | “Quality of Life”[MeSH] |  | Randomized Controlled Trials as Topic[Mesh] |

**S1 Table. Detailed search strategy**

| **POPULATION**  **PubMed**  "Diabetes Mellitus, Type 2"[Mesh] OR Diabetes  "Diabetes Mellitus, Type 2"[Mesh] OR "Diabetes Mellitus, Type 2"[tiab] OR Diabetes[tiab]  **Scopus:**  "Diabetes Mellitus, Type 2" OR Diabetes  **Web of Science:**  "Diabetes Mellitus, Type 2" OR Diabetes  (TI="Diabetes Mellitus, Type 2" OR AB=“Diabetes Mellitus, Type 2”) OR (TI=Diabetes OR AB=Diabetes)  **Cinahl:**  (MM "Diabetes Mellitus, Type 2") OR "Diabetes"  (MM "Diabetes Mellitus, Type 2") OR (TI "Diabetes Mellitus, Type 2" OR AB "Diabetes Mellitus, Type 2") OR (TI diabetes OR AB diabetes)  **PsycInfo:**  exp *Diabetes Mellitus/ OR exp *Type 2 Diabetes/ OR exp *Diabetes/  (Diabetes Mellitus or Type 2 Diabetes or Diabetes).ti,ab.  **Embase:**  'non insulin dependent diabetes mellitus'/exp/mj OR 'diabetes’  'non insulin dependent diabetes mellitus'/exp/mj OR 'non insulin dependent diabetes mellitus':ab,ti OR 'diabetes’:ab,ti |
| --- |
| AND |
| **INTERVENTION**  **PubMed**  "Health Behavior"[Mesh] OR “Health-Related Behavior*” OR “Health Risk Behavior*” OR "Exercise"[Mesh] OR “Physical Activit*” OR "Gymnastics"[Mesh] OR "Yoga"[Mesh] OR Training OR "Running"[Mesh] OR "Jogging"[Mesh] OR "Swimming"[Mesh] OR "Walking"[Mesh] OR "Stair Climbing"[Mesh] OR "Diet"[Mesh] OR "Dietary Approaches To Stop Hypertension"[Mesh] OR "Energy Intake"[Mesh] OR "Caloric Restriction"[Mesh] OR “Fasting”[Mesh] OR “Portion Size”[Mesh] OR “Serving Size”[Mesh] OR “Dietary Restriction*” OR “Dietary Modification*” OR "Healthy Lifestyle"[Mesh] OR "Smoking Cessation"[Mesh] OR Smoking OR "Health Education"[Mesh] OR “Health Literacy” OR “Health Fairs” OR “Health Promotion” OR “Weight Reduction Program*” OR "Self-Management"[Mesh] OR “Blood Glucose Self-Monitoring”[MeSH] OR “Blood Glucose monitor*” OR Self-monitor* OR Medication OR Adherence OR Compliance OR "Diabetes Complications"[Mesh] OR "Cognitive Behavioral Therapy"[Mesh] OR "Psychosocial Intervention"[Mesh]  "Health Behavior"[Mesh] OR "Health Behavior"[tiab] OR “Health-Related Behavior*”[tiab] OR “Health Risk Behavior*”[tiab] OR "Exercise"[Mesh] OR "Exercise"[tiab] OR “Physical Activit*”[tiab] OR "Gymnastics"[Mesh] OR "Gymnastics"[tiab] OR "Yoga"[Mesh] OR "Yoga"[tiab] OR Training[tiab] OR "Running"[Mesh] OR "Running”[tiab] OR "Jogging"[Mesh] OR "Jogging"[tiab] OR "Swimming"[Mesh] OR "Swimming"[tiab] OR "Walking"[Mesh] OR "Walking"[tiab] OR "Stair Climbing"[Mesh] OR "Stair Climbing"[tiab] OR "Diet"[Mesh] OR "Diet"[tiab] OR "Dietary Approaches To Stop Hypertension"[Mesh] OR "Dietary Approaches To Stop Hypertension"[tiab] OR "Energy Intake"[Mesh] OR "Energy Intake"[tiab] OR "Caloric Restriction"[Mesh] OR "Caloric Restriction"[tiab] OR “Fasting”[Mesh] OR “Fasting”[tiab] OR “Portion Size”[Mesh] OR “Portion Size”[tiab] OR “Serving Size”[Mesh] OR “Serving Size”[tiab] OR “Dietary Restriction*”[tiab] OR “Dietary Modification*”[tiab] OR "Healthy Lifestyle"[Mesh] OR "Healthy Lifestyle"[tiab] OR "Smoking Cessation"[Mesh] OR "Smoking Cessation"[tiab] OR “Smoking”[tiab] OR "Health Education"[Mesh] OR "Health Education"[tiab] OR “Health Literacy”[tiab] OR “Health Fairs”[tiab] OR “Health Promotion”[tiab] OR “Weight Reduction Program*”[tiab] OR "Self-Management"[Mesh] OR "Self-Management"[tiab] OR "Blood Glucose Self-Monitoring”[MeSH] OR "Blood Glucose Self-Monitoring”[tiab] OR “Blood Glucose monitor*”[tiab] OR Self-monitor*[tiab] OR Medication[tiab] OR Adherence[tiab] OR Compliance[tiab] OR "Diabetes Complications"[Mesh] OR "Diabetes Complications"[tiab] OR "Cognitive Behavioral Therapy"[Mesh] OR "Cognitive Behavioral Therapy"[tiab] OR "Psychosocial Intervention"[Mesh] OR "Psychosocial Intervention"[tiab]  **Scopus:**  “Health Behavior*” OR “Health-Related Behaviour*” OR “Health Risk Behavior*” OR “Exercise” OR “Physical Activit*” OR “Gymnastics” OR “Yoga” OR Training OR “Running” OR “Jogging” OR “Swimming” OR “Walking” OR “Stair Climbing” OR “Diet” OR “Dietary Approaches To Stop Hypertension” OR “Energy Intake” OR “Caloric Restriction” OR “Fasting” OR “Portion Size” OR “Serving Size” OR “Dietary Restriction*” OR “Dietary Modification*” OR “Healthy Lifestyle” OR “Smoking Cessation” OR “Smoking” OR “Health Education” OR “Health Literacy” OR “Health Fairs” OR “Health Promotion” OR “Weight Reduction Program*” OR “Self-Management” OR “Blood Glucose Self-Monitoring” OR “Blood Glucose monitor*” OR “Self-monitor*” OR “Medication” OR “Adherence” OR “Compliance” OR “Diabetes Complications” OR “Cognitive Behavioral Therapy” OR “Psychosocial Intervention”  **Web of Science:**  “Health Behavior*” OR “Health-Related Behaviour*” OR “Health Risk Behavior*” OR "Exercise” OR “Physical Activit*” OR "Gymnastics” OR "Yoga" OR Training OR "Running" OR "Jogging" OR "Swimming" OR "Walking" OR "Stair Climbing" OR "Diet" OR "Dietary Approaches To Stop Hypertension" OR "Energy Intake" OR "Caloric Restriction” OR “Fasting” OR “Portion Size” OR “Serving Size” OR “Dietary Restriction*” OR “Dietary Modification*” OR "Healthy Lifestyle" OR "Smoking Cessation" OR Smoking OR "Health Education" OR “Health Literacy” OR “Health Fairs” OR “Health Promotion” OR “Weight Reduction Program*” OR "Self-Management" OR “Blood Glucose Self-Monitoring” OR “Blood Glucose monitor*” OR Self-monitor* OR Medication OR Adherence OR Compliance OR "Diabetes Complications" OR "Cognitive Behavioral Therapy" OR "Psychosocial Intervention"  (TI=“Health Behavior*” OR AB=“Health Behavior*”) OR (TI=“Health-Related Behaviour*” OR AB=“Health-Related Behaviour*”) OR (TI=“Health Risk Behavior*” OR AB=“Health Risk Behavior*”) OR (TI=Exercise OR AB=Exercise) OR (TI=“Physical Activit*” OR AB=“Physical Activit*”) OR (TI=Gymnastics OR AB=Gymnastics) OR (TI=Yoga OR AB=Yoga) OR (TI=Training OR AB=Training) OR (TI=Running OR AB=Running) OR (TI=Jogging OR AB=Jogging) OR (TI=Swimming OR AB=Swimming) OR (TI=Walking OR AB=Walking) OR (TI="Stair Climbing" OR AB=“Stair Climbing”) OR (TI=Diet OR AB=Diet) OR (TI="Dietary Approaches To Stop Hypertension" OR AB="Dietary Approaches To Stop Hypertension") OR (TI="Energy Intake" OR AB=“Energy Intake”) OR (TI="Caloric Restriction” OR AB="Caloric Restriction”) OR (TI=Fasting OR AB=Fasting) OR (TI=“Portion Size” OR AB=“Portion Size”) OR (TI=“Serving Size” OR AB=“Serving Size”) OR (TI=“Dietary Restriction*” OR AB=“Dietary Restriction*”) OR (TI=“Dietary Modification*” OR AB=“Dietary Modification*”) OR (TI="Healthy Lifestyle" OR AB="Healthy Lifestyle") OR (TI="Smoking Cessation" OR AB="Smoking Cessation") OR (TI=Smoking OR AB=Smoking) OR (TI="Health Education" OR AB=“Health Education") OR (TI=“Health Literacy” OR AB=“Health Literacy”) OR (TI=“Health Fairs” OR AB=“Health Fairs”) OR (TI=“Health Promotion” OR AB=“Health Promotion”) OR (TI=“Weight Reduction Program*” OR AB=“Weight Reduction Program*”) OR (TI="Self-Management" OR AB="Self-Management") OR (TI=“Blood Glucose Self-Monitoring” OR AB=“Blood Glucose Self-Monitoring”) OR (TI=“Blood Glucose monitor*” OR AB=“Blood Glucose monitor*”) OR (TI=Self-monitor* OR AB= Self-monitor*) OR (TI=Medication OR AB=Medication) OR (TI=Adherence OR AB=Adherence) OR (TI=Compliance OR AB=Compliance) OR (TI="Diabetes Complications" OR AB="Diabetes Complications") OR (TI="Cognitive Behavioral Therapy" OR AB=“Cognitive Behavioral Therapy") OR (TI="Psychosocial Intervention" OR AB=“Psychosocial Intervention")  **Cinahl:**  (MH "Health Behavior+") OR “Health-Related Behaviour*” OR “Health Risk Behavior*” OR (MH "Exercise") OR (MH "Physical Activity") OR (MH "Gymnastics") OR (MH "Yoga+") OR “Training” OR (MH "Running+") OR (MH "Jogging") OR (MH "Swimming") OR (MH "Walking+") OR (MH "Stair Climbing") OR (MH "Diet+") OR (MH "DASH Diet") OR (MH "Energy Intake") OR "Caloric Restriction" OR (MH "Fasting") OR (MH "Portion Size") OR "Serving Size" OR "Dietary Restriction*" OR “Dietary Modification*” OR "Healthy Lifestyle" OR (MH "Smoking Cessation") OR (MH “Smoking”) OR (MH "Health Education") OR (MH “Health Literacy”) OR (MH "Health Fairs") OR (MH "Health Promotion+") OR (MH "Weight Reduction Programs") OR (MH "Self-Management") OR (MH "Blood Glucose Monitoring+") OR "Self-monitor*" OR "Medication" OR "Adherence" OR "Compliance" OR "Diabetes Complications" OR (MH "Cognitive Therapy+") OR (MH "Psychosocial Intervention")  (MH "Health Behavior+") (TI “Health Behaviour” OR AB “Health Behaviour”) OR (TI “Health-Related Behaviour*” OR AB “Health-Related Behaviour*”) OR (TI “Health Risk Behavior*” OR AB “Health Risk Behavior*”) OR (TI Exercise OR AB Exercise) OR (TI "Physical Activity" OR AB "Physical Activity”) OR (TI Gymnastics OR AB Gymnastics) OR (TI Yoga OR AB Yoga) OR (TI Training OR AB Training) OR (TI Running OR AB Running) OR (TI Jogging OR AB Jogging) OR (TI Swimming OR AB Swimming) OR (TI Walking OR AB Walking) OR (TI “Stair Climbing” OR AB “Stair Climbing”) OR (TI Diet OR AB Diet) OR (TI “DASH Diet" OR AB “DASH Diet”) OR (TI "Energy Intake" OR AB “Energy Intake”) OR (TI "Caloric Restriction" OR AB "Caloric Restriction") OR (TI Fasting OR AB Fasting) OR (TI "Portion Size" OR AB “Portion Size”) OR (TI "Serving Size" OR AB "Serving Size") OR (TI "Dietary Restriction*" OR AB “Dietary Restriction*") OR (TI “Dietary Modification*” OR AB “Dietary Modification*”) OR (TI "Healthy Lifestyle" OR AB “Healthy Lifestyle") OR (TI "Smoking Cessation" OR AB "Smoking Cessation") OR (TI Smoking OR AB Smoking) OR (TI "Health Education" OR AB "Health Education") OR (TI “Health Literacy” OR AB “Health Literacy”) OR (TI "Health Fairs" OR AB "Health Fairs") OR (TI "Health Promotion" OR AB "Health Promotion”) OR (TI "Weight Reduction Program*" OR AB "Weight Reduction Program*") OR (TI "Self-Management" OR AB "Self-Management") OR (TI "Blood Glucose Monitoring" OR AB "Blood Glucose Monitoring") OR (TI "Self-monitor*" OR AB "Self-monitor*") OR (TI Medication OR AB Medication) OR (TI Adherence OR AB Adherence) OR (TI Compliance OR AB Compliance) OR (TI “Diabetes Complication*" OR AB “Diabetes Complication*") OR (TI "Cognitive Behavioral Therapy" OR AB “Cognitive Behavioral Therapy”) OR (TI "Psychosocial Intervention" OR AB “Psychosocial Intervention”)  **PsycInfo:**  exp Health Behavior/ OR Health-Related Behavior.mp. OR *health risk behavior/ OR exp *Exercise/ OR exp *Physical Activity/ OR Gymnastics.mp. OR exp *Yoga/ OR exp *Training/ OR *Running/ OR Jogging.mp. OR *Swimming/ OR *Walking/ OR Stair Climbing.mp. OR *Diets/ OR Dietary Approaches To Stop Hypertension.mp. OR Energy Intake.mp. OR Caloric Restriction.mp. OR Fasting.mp. OR Portion Size.mp. OR Serving Size.mp. OR Dietary Restriction.mp. OR Dietary Modification.mp. OR Healthy Lifestyle.mp. OR exp *Smoking Cessation/ OR Smoking.mp. OR Health Education/ OR Health Literacy/ OR Health Fairs.mp. OR Health Promotion/ OR Weight Reduction Programs.mp. OR exp *Self-Management/ OR exp *Self-Monitoring/ or Blood Glucose Monitoring.mp. OR Medication.mp. OR exp *Treatment Compliance/ OR Adherence.mp. OR Diabetes Complications.mp. OR exp *Cognitive Behavior Therapy/ OR Psychosocial Intervention.mp.  (Health Behavior* OR Health-Related Behaviour* OR Health Risk Behavior* OR Exercise OR Physical Activit* OR Gymnastics OR Yoga OR Training OR Running OR Jogging OR Swimming OR Walking OR Stair Climbing OR Diet OR Dietary Approaches To Stop Hypertension OR Energy Intake OR Caloric Restriction OR Fasting OR Portion Size OR Serving Size OR Dietary Restriction* OR Dietary Modification* OR Healthy Lifestyle OR Smoking Cessation OR Smoking OR Health Education OR Health Literacy OR Health Fairs OR Health Promotion OR Weight Reduction Program* OR Self-Management OR Self-Monitoring or Blood Glucose Monitoring OR Medication OR Treatment Compliance OR Adherence OR Diabetes Complications OR Cognitive Behavior Therapy OR Psychosocial Intervention).ti,ab.  **Embase:**  'health behavior'/exp/mj OR 'high risk behavior'/exp OR 'exercise'/exp/mj OR 'physical activity'/exp/mj OR 'gymnastics'/exp/mj OR 'yoga'/exp OR 'training'/exp/mj OR 'running'/exp/mj OR 'jogging'/exp/mj OR 'swimming'/exp/mj OR 'walking'/exp/mj OR 'stair climbing'/exp/mj OR 'diet'/exp/mj OR 'DASH diet'/exp/mj OR 'caloric intake'/mj OR 'caloric restriction'/mj OR 'fasting'/mj OR 'portion size'/exp/mj OR 'diet restriction'/mj OR 'dietary modification'/mj OR 'healthy lifestyle'/mj OR 'smoking cessation'/mj OR 'smoking'/mj OR 'health education'/mj OR 'health literacy'/mj OR 'health promotion'/exp/mj OR 'weight loss program'/mj OR 'self care'/exp/mj OR 'blood glucose monitoring'/mj OR 'self monitoring'/mj OR 'medication' OR 'adherence'/mj OR 'medication compliance'/mj OR 'diabetic complication'/exp/mj OR 'cognitive therapy'/exp/mj OR 'psychosocial intervention'/mj  'health behavior'/exp/mj OR 'health behavior':ab,ti OR 'high risk behavior':ab,ti OR 'exercise':ab,ti OR 'physical activity':ab,ti OR 'gymnastics':ab,ti OR 'yoga':ab,ti OR 'training':ab,ti OR 'running':ab,ti OR 'jogging':ab,ti OR 'swimming':ab,ti OR 'walking':ab,ti OR 'stair climbing':ab,ti OR 'diet':ab,ti OR 'DASH diet':ab,ti OR 'caloric intake':ab,ti OR 'caloric restriction':ab,ti OR 'fasting':ab,ti OR 'portion size':ab,ti OR 'diet restriction’:ab,ti OR 'dietary modification':ab,ti OR 'healthy lifestyle':ab,ti OR 'smoking cessation':ab,ti OR 'smoking':ab,ti OR 'health education':ab,ti OR 'health literacy':ab,ti OR 'health promotion':ab,ti OR 'weight loss program':ab,ti OR 'self care':ab,ti OR 'blood glucose monitoring':ab,ti OR 'self monitoring':ab,ti OR 'medication':ab,ti OR 'adherence':ab,ti OR 'medication compliance':ab,ti OR 'diabetic complication':ab,ti OR 'cognitive therapy':ab,ti OR 'psychosocial intervention':ab,ti |
| AND |
| **OUTCOME**  **PubMed**  “Quality Of Life”[Mesh] OR “Life Quality” OR “Health Related Quality Of Life” OR HRQOL OR “Short form questionnaire*” OR EuroQOL OR WHOQOL OR DQOL OR ADDQOL OR Well-being OR “Diabetes Health Profile” OR “Sickness Impact Profile”  “Quality Of Life”[Mesh] OR “Quality Of Life”[tiab] OR “Life Quality”[tiab] OR “Health Related Quality Of Life”[tiab] OR HRQOL[tiab] OR “Short form questionnaire*”[tiab] OR EuroQOL[tiab] OR WHOQOL[tiab] OR DQOL[tiab] OR ADDQOL[tiab] OR Well-being[tiab] OR “Diabetes Health Profile”[tiab] OR “Sickness Impact Profile”[tiab]  **Scopus:**  “Quality Of Life” OR “Life Quality” OR “Health Related Quality Of Life” OR HRQOL OR “Short form questionnaire*” OR EuroQOL OR WHOQOL OR DQOL OR ADDQOL OR Well-being OR “Diabetes Health Profile” OR “Sickness Impact Profile”  **Web of Science:**  “Quality Of Life” OR “Life Quality” OR “Health Related Quality Of Life” OR HRQOL OR “Short form questionnaire*” OR EuroQOL OR WHOQOL OR DQOL OR ADDQOL OR Well-being OR “Diabetes Health Profile” OR “Sickness Impact Profile”  (TI=“Quality Of Life” OR AB=“Quality Of Life”) OR (TI=“Life Quality” OR AB=“Life Quality”) OR (TI=“Health Related Quality Of Life” OR AB=“Health Related Quality Of Life”) OR (TI=HRQOL OR AB=HRQOL) OR (TI=“Short form questionnaire*” OR AB=“Short form questionnaire*”) OR (TI=EuroQOL OR AB=EuroQOL) OR (TI=WHOQOL OR AB=WHOQOL) OR (TI=DQOL OR AB=DQOL) OR (TI=ADDQOL OR AB=ADDQOL) OR (TI=Well-being OR AB=Well-being) OR (TI=“Diabetes Health Profile” OR AB=“Diabetes Health Profile”) OR (TI=“Sickness Impact Profile” OR AB=“Sickness Impact Profile”)  **Cinahl:**  (MH "Quality of Life+") OR "Life Quality" OR "Health Related Quality Of Life" OR "HRQOL" OR "Short form questionnaire*" OR "EuroQOL" OR “WHOQOL” OR "DQOL" OR "ADDQOL" OR "Well-being" OR "Diabetes Health Profile" OR (MH "Sickness Impact Profile")  (MH "Quality of Life+") OR (TI "Quality of Life" OR AB "Quality of Life") OR (TI "Life Quality" OR AB “Life Quality”) OR (TI "Health Related Quality Of Life" OR AB "Health Related Quality Of Life") OR (TI "HRQOL" OR AB “HRQOL”) OR (TI "Short form questionnaire*" OR AB "Short form questionnaire*") OR (TI EuroQOL OR AB EuroQOL) OR (TI WHOQOL OR AB WHOQOL) OR (TI DQOL OR AB DQOL) OR (TI ADDQOL OR AB ADDQOL) OR (TI Well-being OR AB Well-being) OR (TI “Diabetes Health Profile" OR AB “Diabetes Health Profile") OR (TI "Sickness Impact Profile" OR AB "Sickness Impact Profile")  **PsycInfo:**  exp "Quality of Life"/ OR Life Quality.mp. OR exp "Health Related Quality of Life"/ OR HRQOL.mp. OR Short form questionnaire.mp. OR EuroQOL.mp. OR WHOQOL.mp. OR DQOL.mp. OR ADDQOL.mp. OR Well Being/ OR Diabetes Health Profile.mp. OR Sickness Impact Profile.mp.  (Quality of Life OR Life Quality OR Health Related Quality of Life OR HRQOL OR Short form questionnaire OR EuroQOL OR WHOQOL OR DQOL OR ADDQOL OR Well Being OR Diabetes Health Profile OR Sickness Impact Profile).ti,ab.  **Embase:**  'quality of life'/exp/mj OR 'Short Form 36'/exp/mj OR 'European Quality of Life 5 Dimensions questionnaire'/exp/mj OR ‘WHOQOL-100’/exp/mj OR 'diabetes quality of life questionnaire'/mj OR 'audit of diabetes dependent quality of life'/mj OR 'wellbeing'/exp/mj OR 'Sickness Impact Profile'/mj  'quality of life'/exp/mj OR 'quality of life':ab,ti OR 'Short Form 36':ab,ti OR 'European Quality of Life 5 Dimensions questionnaire':ab,ti OR ‘WHOQOL-100’: ab,ti OR 'diabetes quality of life questionnaire':ab,ti OR 'audit of diabetes dependent quality of life':ab,ti OR 'wellbeing':ab,ti OR 'Sickness Impact Profile':ab,ti |
| AND |
| **STUDY DESIGN**  **PubMed:**  "Randomized Controlled Trials as Topic"[Mesh] OR "Randomized Controlled Trial"[Publication Type] OR "Clinical Trials as Topic"[Mesh] OR "Clinical Trial"[Publication Type] OR "Controlled Clinical Trials as Topic"[Mesh] OR “Randomized”  "Controlled Clinical Trials as Topic"[Mesh] OR "Controlled Clinical Trial*"[tiab] OR "Clinical Trials as Topic"[Mesh] OR "Clinical Trial*"[tiab] OR "Clinical Trial"[Publication Type] OR "Randomized"[tiab] OR "Randomized Controlled Trials as Topic"[Mesh] OR "Randomized Controlled Trial"[Publication Type] OR "Randomized Controlled Trial*"[tiab]  **Scopus:**  “Clinical Trial” OR “Controlled Clinical Trial” OR “Randomized” OR “Randomized Controlled Trial”  **Web of Science:**  (TI=“Clinical Trial” OR AB=“Clinical Trial”) OR (TI=“Controlled Clinical Trial” OR AB=“Controlled Clinical Trial”) OR (TI=Randomized OR AB=Randomized) OR (TI=“Randomized Controlled Trial” OR AB=“Randomized Controlled Trial”)  **CINAHL:**  (MM "Clinical Trials+") OR (MH "Randomized Controlled Trials+") OR "Controlled clinical trial" OR "Randomized"  (MM "Clinical Trials+") OR (TI “Clinical Trials” OR AB “Clinical Trials”) OR (MH "Randomized Controlled Trials+") OR (TI “Randomized Controlled Trials” OR AB “Randomized Controlled Trials”) OR (TI "Controlled clinical trial" OR AB "Controlled clinical trial") OR (TI Randomized OR AB Randomized)  **Psycinfo:**  exp *Clinical Trials/ OR controlled clinical trial.mp. OR exp *Randomized Controlled Trials/ OR randomized.mp.  (Clinical Trials OR controlled clinical trial OR Randomized Controlled Trials OR randomized).ti,ab.  **Embase:**  'clinical trial'/exp/mj OR 'randomized controlled trial'/mj OR 'controlled clinical trial'/mj  'clinical trial':ab,ti OR 'controlled clinical trial':ab,ti OR 'randomized controlled trial':ab,ti OR 'randomized':ab,ti |
