# Supplementary material for: The effect of health behavior interventions to manage Type 2 diabetes on the quality of life in low-and middle-income countries: A systematic review and meta-analysis
Source: PLoS One. 2023 Oct 16;18(10):e0293028. doi: 10.1371/journal.pone.0293028 (PMC10578590; doi:10.1371/journal.pone.0293028)
Supplement: S2 Table — (DOCX) [file pone.0293028.s007.docx]

**S2 Table. Intervention characteristics of studies included in the review sorted alphabetically by author**

| **Author (Year)** | **Intervention description** | **Intervention duration** | **Intervention delivered by** | **Delivery setting** |
| --- | --- | --- | --- | --- |
| Abraham (2020) [47] | Problem solving, relaxation, stress reduction | 4 individual SME sessions 30-45 min long held fortnightly, follow up at 3 months post-intervention | AHP | Hospital |
| Akinci (2018) [67] | PA | 3 * 50-60 min aerobic and resistance exercises per week for 8 weeks | AHP | Hospital |
| Anderson (2009) [51] | Diet, glucose monitoring | One-to-one meeting between participant and the self-management consultant at baseline, one year and two years, with monthly telephone follow-up in between | HCP and AHP | University department/health centre |
| Arora (2009) [44] | PA | 2 * progressive resistance training per week consisting of 5 min warm up, followed by seven exercises for muscle groups and 5 min of cool down. Aerobic exercise group performed walking for 30 mins per day, 3 times a week for 8 weeks. | Exercise trainers | University department/health centre |
| Arovah (2018) [55] | PA | Pedometer, workbook consisting of social cognitive theory-based activities and text messages 1-3 times a day for 12 weeks (1st phase), and maintenance of 1st phase activities for the next 12 weeks (2nd phase) without text message support | AHP | Home |
| Azami (2018) [8] | PA, diet, medication, glucose monitoring, preventing complications such as foot care | 4 * 10 min movie clips, 4 * 120 min group discussion sessions, weekly telephone follow up calls 2 months after the end of group discussions (altogether 12 weeks of intervention). F/up at 3 months and 6 months | HCP | Diabetes clinic |
| Browning (2016) [66] | PA, diet, medication, glucose monitoring, preventing complications such as foot care | 3 * 2 face-to-face and 2 telephone coaching per month, frequency of intervention decreased over the 12-month intervention such that, participants received 1 face-to-face and 1 telephone session per month in the last 6 months period | HCP and AHP | CHC |
| Butt (2015) [60] | PA, diet, medication, glucose monitoring, preventing complications | 35-45 min education session at enrolment, 20-30 min intervention reinforcement on 2nd visit at 3 months, follow up at 6 months | HCP | Diabetes clinic |
| Cani (2015) [70] | PA, diet, medication, glucose monitoring, smoking cessation, preventing complications such as foot care | Monthly individual appointment with clinical pharmacist for 6 months | HCP | Diabetes clinic |
| Castillo-Hernandez (2020) [61] | PA, diet, medication, glucose monitoring, preventing complications | 16 * 1-hour weekly SME for both intervention and control groups. All study subjects completed individual nutrition counseling during the first month and were motivated to attend, 2 * 50 min exercise sessions per week for 8 months. 20 peer-support meetings for 8 months (1 session every week, except for 2 holiday weeks and a 1-month Chikungunya outbreak) per group of 3-4 participants. | PL/LP | CHC |
| Chaveepojnkamjorn (2009) [65] | PA, diet, glucose monitoring, problem solving | 5 * 2-hr monthly session in small groups for 16 weeks, follow up data collected at 12 weeks and 24 weeks | HCP | CHC |
| Cheng (2019) [62] | Diet, glucose monitoring, problem solving, | a brief intake session, 2 * weekly face-to-face small group discussion sessions and 4 * weekly phone-based individualized consultation and maintenance sessions, follow up at 1 week and 3 months after intervention | HCP | Hospital |
| Dede (2015) [75] | PA, diet | 3 times a week exercise on a treadmill slowly increasing duration and intensity over the period of 12 weeks | Exercise trainers | Not stated |
| Ebrahimi (2018) [10] | PA, diet, medication, glucose monitoring, preventing complications | 3 * 1.5-hr training session for 3 weeks and telephone follow up every 2 weeks for 12 weeks | Author | Diabetes clinic |
| Jaipakdee (2015) [68] | PA, diet, medication, glucose monitoring, preventing complications, foot care, stress reduction, problem solving | 6 * 3-hr long monthly sessions | HCP | CHC |
| Jamshidpour (2020) [46] | PA | 3/week exercise sessions for 8 weeks consisting of warming up, 20-45 min of static cycling, lower extremity resistance exercise training and cool down. | AHP | Hospital |
| Kong (2019) [72] | PA, diet, medication, glucose monitoring, smoking cessation, preventing complications such as foot care | Five components of CCM-based intervention that included awareness of diabetes management, self-management support strategies, decision support, and clinical information system | HCP | CHC |
| Lyu (2021) [57] | PA, diet, medication, glucose monitoring | “Internet +” transitional care platform for 3 months | HCP | Hospital |
| Maharaj (2015) [37] | PA | 3 * 15 min exercise per week for the first 4 weeks, 3 * 20 min per week in the next 4 weeks, and 3 * 30 min per week in the final 4 weeks | AHP | Hospital |
| Mash (2014) [56] | PA, diet, medication, glucose monitoring, smoking cessation, preventing complications such as foot care | 4 * 60 min sessions | PL/LP | CHC |
| Mohammadi (2018) [36] | PA, diet, medication, glucose monitoring, preventing complications | 8 * 2-hr session for 12-weeks, follow-up done at 24-week post intervention | Not stated | Diabetes clinic |
| Nazir (2020) [54] | Medication | 2 * 15 min education intervention in a month for 12 weeks | HCP | Hospital |
| Nouripour (2021) [52] | Diet | 40%– 45% of total carbohydrate and total protein intake provided at dinner and evening snack to high-carbohydrate group and high-protein group respectively for 10 weeks | AHP | Outpatient clinic |
| Peimani (2018) [49] | Experience/story sharing, PA, diet, medication, stress reduction, problem solving | 2-hr monthly peer support meeting for 6 months along with weekly telephone contacts between participants and peer supporters | PL/LP | Diabetes clinic |
| Rasoul (2019) [11] | PA, diet, medication, glucose monitoring | 1.3-hr education, 3 days a week for 20 weeks | Web-based, not stated by who | Diabetes clinic |
| Rias (2020) [48] | PA | 30 min of walk, 5 times/week for 8 weeks 2L/day of alkaline electrolysed water intake for 8 weeks | PL/LP | Home |
| Rondhianto (2018) [38] | Self-care behavior | 6 * 2 hr long education session (1st and last sessions were home visit sessions and the rest were group sessions) | Not stated | CHC |
| Safavi (2011) [73] | PA, diet, medication, glucose monitoring, smoking cessation, preventing complications such as foot care, stress reduction | 45 min of weekly education sessions for 6 weeks | HCP | Hospital |
| Saghaee (2020) [41] | PA, diet, medication, glucose monitoring, preventing complications, stress reduction, problem solving | 8 * 2.5-hr educational workshops over 4 weeks | HCP | Outpatient clinic |
| Sekhar (2019) [43] | Medication, preventing/managing complication | 20-25 min patient interview and counselling, followed by 6 * monthly telephone contacts | HCP | Hospital |
| Shahsavari (2021) [45] | PA, diet, problem solving | 2 hr education session for 3 months and 15-20 min telephone contact once a week | PL/LP | Diabetes clinic |
| Shenoy (2009) [35] | PA | Regular walk, 5 days a week for 8 weeks using HRM and pedometer | Not stated | University department/health centre |
| Shi (2018) [71] | PA, diet, stress reduction | 3 times western medicine education and 3 times integrative education in the first 3 months with follow up at 3, 6, 9 and 12 months | HCP | Hospital |
| Singh (2020) [40] | PA, diet | Daily yoga session for 2 weeks and 3 months of self-practice | Yoga instructors | Hospital |
| Sreedevi (2017) [39] | PA, diet, medication, stress reduction, preventing complications | 2 * 60 min yoga sessions consisting of Surya namaskara, deep relaxation-muscle relaxation technique, yoga postures and pranayama. 3 * 45-60 min peer support session with telephone follow up for those allocated in peer intervention group. | Yoga instructors, PL/LP | CHC |
| Sunil (2020) [53] | Diet, medication, glucose monitoring | Use of mobile phone application named ‘Diaguru’ for six months | Web-based, not stated by who | Hospital |
| Tapehsari (2020) [50] | PA, diet | 150 min moderate intensity physical activity per week. SME on healthy diet and physical activity | HCP | Diabetes clinic |
| Torabizadeh (2018) [42] | Problem solving, experience/story sharing | 6 * weekly 1.5-hr meeting, follow up after 3 months | Author | Outpatient clinic |
| Umphonsathien (2022) [64] | Diet | 600 kcal/day diet * 10 days for 2-week run-in period, 600 kcal/day diet for 2 or 4 non-consecutive days/week for 18-week intermittent caloric restriction days | Not stated | Hospital |
| Wattana (2007) [58] | PA, diet, medication, preventing complications such as foot care, stress reduction | 120 min group diabetes education class, 4 * 90 min/group discussions, 2 * 45 min home visits from the researchers | HCP | Diabetic clinic |
| Wichit (2017) [9] | PA, diet, problem solving, preventing complications, | 3 * 2-hr education session | HCP | Diabetes clinic |
| Wongrochananan (2015) [69] | PA, diet, medication, glucose monitoring, foot care | 3 months IMM intervention | Web-based, not stated by who | Government offices |
| Yang (2022) [74] | PA, diet | Reminders to upload daily meals and snacks, weight, fasting glucose, blood pressure and medication in the “The home of Xinqiao nutrition” platform in WeChat and monthly 40-min telephone education for 12 months | Web-based, not stated by who | CHC |
| Yucel (2015) [63] | PA | 3 * 45-70 mins long weekly exercise for 12 weeks | AHP | Hospital |
| Zuo (2020) [59] | PA, meditation, sleep monitoring | 7 * 40-50 min session, followed by 10-15 min discussion for 2 months, follow up 6 months after intervention | HCP | CHC |

PA: Physical activity, HCP: Health care professionals, AHP: Allied health professionals, PS/LP: Peer supporters/lay people, CHC: Community health centres
