# Supplementary material for: The effect of health behavior interventions to manage Type 2 diabetes on the quality of life in low-and middle-income countries: A systematic review and meta-analysis
Source: PLoS One. 2023 Oct 16;18(10):e0293028. doi: 10.1371/journal.pone.0293028 (PMC10578590; doi:10.1371/journal.pone.0293028)
Supplement: S3 Table — (DOCX) [file pone.0293028.s008.docx]

**S3 Table. Effect of health behavior intervention on the primary (quality of life) and secondary outcomes**

| **Author (Year)** | **Outcome type** | **Outcome measures** | **Duration between pre- and post-intervention assessments** | **Mean Pre-intervention value (SD)** | **Mean Post-intervention value (SD)** | **Change in value** | **Lower 95% confidence interval** | **Upper 95% confidence interval** | ***P*-value** | **Effect size** |
| --- | --- | --- | --- | --- | --- | --- | --- | --- | --- | --- |
| Mash (2014) [56] | QOL | Physical functioning | 52 weeks | 74.8 (21.3) | 26.4 (6.1) | - | - | - | 0.668 | - |
|  |  | Role functioning |  | 79.2 (26.5) | 81.7 (25.6) | - | - | - | 0.503 | - |
|  |  | Social functioning |  | 65.9 (32.2) | 63.2 (30.8) | - | - | - | 0.95 | - |
|  |  | Mental health |  | 59.3 (14.3) | 60.1 (13.7) | - | - | - | 0.966 | - |
|  |  | General health |  | 56.5 (13.9) | 58.8 (10.8) | - | - | - | 0.179 | - |
|  |  | Pain |  | 57.9 (28.1) | 57.7 (29.9) | - | - | - | 0.984 | - |
|  | Glycemic | HbA1c (%) |  | 8.9 (2.3) | 8.4 (2.0) | - | - | - | 0.967 | - |
|  | Weight management | Body weight (kg) |  | 84.4 (18.7) | 83.8 (20.2) | - | - | - | 0.392 | - |
|  | Self-care behaviors | Physical activity days/week |  | 3.5 (2.4) | 3.9 (2.3) | - | - | - | 0.574 | - |
|  |  | Diet plan days/week |  | 4.0 (2.2) | 4.6 (2.1) | - | - | - | 0.802 | - |
|  |  | Medication days/week |  | 6.7 (1.2) | 6.8 (0.8) | - | - | - | 0.897 | - |
|  |  | Foot care days/week |  | 4.7 (2.6) | 5.5 (2.1) | - | - | - | 0.38 | - |
|  |  | Smoking (%) |  | 130/710 (18.3) | 78/409 (19.1) | - | - | - | 0.8 | - |
| Azami (2018) [8] | QOL | Mean QOL | 26 weeks | 50.42 (9.17) | 50.67 (9.04) | - | - | - | **P<0001** | 0.07 |
|  | Glycemic | HbA1c (%) |  | 9.3 (1.06) | 7.9 (0.93) | - | - | - | P<0001 | 0.67 |
|  | Anthropometric | Weight |  | 82.58 (11.08) | 82 (10.89) | - | - | - | 0.01 | 0.04 |
|  |  | BMI (kg/m^2^) |  | 28.69 (3.2) | 28.55 (3.2) | - | - | - | 0.04 | 0.02 |
|  | Self-care behaviors |  |  | 3.56(1.22) | 5.41(1.15) |  |  |  | P<0001 | 0.61 |
| Lyu (2021) [57] | QOL | Mean QOL | 13 weeks | 554.53 (79.05) | 660.86 (46.5) | - | - | - | **P<0.01** | 0.52 |
|  | Glycemic | HbA1c (%) |  | 7.69 (1.60) | 7.33 (1.42) | - | - | - | P<0.01 | 0.25 |
|  |  | Self-efficacy |  | 6.57 (1.47) | 8.15 (2.76) | - | - | - | P<0.01 | 0.5 |
|  |  | Treatment adherence |  | 3.29 (0.71) | 4.28 (0.75) | - | - | - | P<0.01 | 0.73 |
| Wattana (2007) [58] | QOL | Mean QOL | 24 weeks | 60.61 (15.27) | 70.43 (14.70) | - | - | - | **0** |  |
|  | Glycemic | HbA1c |  | 8.08 (1.87) | 7.40 (1.25) | - | - | - | 0.014 |  |
| Shenoy (2009) [35] | Well-being | General well-being | 8 weeks | 22.5 (5.5) | 29.0 (3.8) | - | - | - | **0.0001** |  |
|  | Glycemic | HbA1c (%) |  | 7.25 (1.0) | 6.5 (0.87) | - | - | - | 0.0001 |  |
|  |  | FBG (mg/dl) |  | 181.0 (40) | 104.6 (14.7) | - | - | - | 0.0001 |  |
|  | Anthropometric | BMI |  | 27.6 (2.9) | 26.5 (2.8) | - | - | - | 0.0001 |  |
| Mohammadi (2018) [36] | QOL | Total DQOL | 36 weeks | 52.8 (3.42) | 57.6 (3.99) | - | - | - | **0.001** |  |
|  | Glycemic | HbA1c (%) |  | 7.97(1.01) | 7.29(0.66) | - | - | - | 0.001 |  |
|  |  | FBG |  | 9.7 (1.88) | 7.6 (0.68) | - | - | - | 0.001 |  |
|  | Anthropometric | BMI |  | 27.1 (1.92) | 25.7 (1.77) | - | - | - | 0.001 |  |
|  |  | Body weight |  | 72.2 (7.84) | 68.3 (6.81) | - | - | - | 0.001 |  |
| Wichit (2017) [9] | QOL | PCS | 13 weeks | 46.7 (6.6) | 49.9 (6.9) | - | - | - | **0.04** |  |
|  |  | MCS |  | 54.1 (8.6) | 58.4 (7.2) | - | - | - | **0.03** |  |
|  | Glycemic | HbA1c (%) |  | 7.0 (2.0) | 7.0 (1.2) | - | - | - | 0.3 |  |
|  |  | Self-management |  | 80.9 (15.9) | 102.8 (12.1) | - | - | - | <0.001 |  |
| Zuo (2020) [59] | QOL | QOL score | 26 weeks | 54.5(14.0) | 43.8(9.4) | - | 0.78 | 1.25 | **<0.001** | 0.91 |
|  |  | Physiological dimension |  | 26.7(8.2) | 21.1(7.1) | - | - | - | <0.001 |  |
|  |  | Psychol/spiritual dimension |  | 16.4(4.4) | 13.0(4.4) | - | - | - | <0.001 |  |
|  |  | Social dimension |  | 6.2(2.6) | 4.5(1.5) | - | - | - | <0.001 |  |
|  |  | Therapeutic dimension |  | 5.3(1.7) | 5.2(1.6) | - | - | - | 0.07 |  |
|  | Glycemic | HbA1c (%) |  | 8.1(1.9) | 7.1(1.1) | - | - | - | <0.001) |  |
| Butt (2015) [60] | QOL | Mean score | 26 weeks | 75.1 (16.6) | 82.45 (10.3) | - | - | - | **0.007** |  |
|  |  | Mobility |  | 14 (42.4%) | 6 (18.2%) | - | - | - | 0.03 |  |
|  |  | Selfcare |  | 2 (6.1%) | 0 | - | - | - | 0.15 |  |
|  |  | Usual activities |  | 3 (9.1%) | 1(3%) | - | - | - | 0.3 |  |
|  |  | Pain/discomfort |  | 14 (42.4%) | 9 (27.3%) | - | - | - | 0.19 |  |
|  |  | Anxiety/depression |  | 12 (36.4%) | 0 | - | - | - | 0.0001 |  |
|  | Glycemic | HbA1c (%) |  | 9.7 (1.57) | 8.5 (1.61) | - | - | - | 0.001 |  |
|  | Anthropometric | BMI |  | 29.3 (5.22) | 28.9 (5.16) | - | - | - | 0.03 |  |
| Maharaj (2015) [37] | QOL | Physical functioning | 12 weeks | 76.8 (18.6) | 91.0 (12.3) | - | - | - | **<0.05** |  |
|  |  | Role physical |  | 63.5 (22.7) | 84.5 (19.5) | - | - | - | <0.05 |  |
|  |  | Role emotional |  | 62.7 (31.3) | 67.7 (27.1) | - | - | - | 0.045 |  |
|  |  | Vitality |  | 57.0 (16.5) | 64.6 (22.4) | - | - | - | <0.05 |  |
|  |  | Mental health |  | 65.3 (19.5) | 69.6 (21.0) | - | - | - | <0.05 |  |
|  |  | Social functioning |  | 53.7 (25.9) | 61.4 (26.3) | - | - | - | <0.05 |  |
|  |  | Bodily pain |  | 55.0 (13.1) | 60.9 (17.5) | - | - | - | 0.026 |  |
|  |  | General health |  | 44.8 (17.9) | 56.0 (15.1) | - | - | - | 0.036 |  |
|  | QOL | Physical functioning |  | 84.2 (17.7) | 93.0 (10.7) | - | - | - | **<0.05** |  |
|  |  | Role physical |  | 63.0 (28.2) | 72.0 (28.4) | - | - | - | <0.05 |  |
|  |  | Role emotional |  | 70.2 (24.5) | 71.8 (24.9) | - | - | - | 0.045 |  |
|  |  | Vitality |  | 61.0 (15.7) | 64.6 (18.2) | - | - | - | <0.05 |  |
|  |  | Mental health |  | 67.7 (20.4) | 76.0 (21.4) | - | - | - | <0.05 |  |
|  |  | Social functioning |  | 57.8 (17.6) | 71.2 (21.3) | - | - | - | <0.05 |  |
|  |  | Bodily pain |  | 52.5 (12.4) | 56.3 (14.5) | - | - | - | 0.026 |  |
|  |  | General health |  | 44.5 (12.5) | 54.3 (10.9) | - | - | - | 0.036 |  |
| Castillo-Hernandez (2020) [61] | QOL | Energy and mobility (%) | 35 weeks | 45.9 |  | -14.5 (28.1) | - | - | 0.03 |  |
|  |  | Diabetes control (%) |  | 49.5 |  | -31.3 (27.2) | - | - | 0.02 |  |
|  |  | Anxiety and worry (%) |  | 62.5 |  | -36.2 (25.4) | - | - | 0.06 |  |
|  |  | Social burden (%) |  | 30.8 |  | -22.5 (13.3) | - | - | 0.35 |  |
|  |  | Sexual functioning (%) |  | 27.7 |  | -18.4 (49.6) | - | - | 0.17 |  |
|  |  | Summary scores (%) |  | 53.6 |  | -27.6 (29.3) | - | - | **0.03** |  |
|  | Glycemic | HbA1c (%) |  | 8.8 (2.2) |  | -1.29 (1.97) | - | - | 0.32 |  |
|  | Anthropometric | BMI |  | 29.2 (5) |  | -0.9 (0.8) | - | - | 0.65 |  |
|  | Self-management | Physical activity (METs/min per week) |  | 2,575 (2,695) |  | 1,073 (4045) | - | - | 0.19 |  |
| Rondhianto (2018) [38] | QOL | QOL score | 18 weeks | 66.0 (17.09) | 92.3 (11.17) | - | - | - | **0.01** |  |
|  | Glycemic | Blood glucose level (mg/dl) |  | 207.6 (63.69) | 118.3 (23.50) | - | - | - | 0.001 |  |
|  | Self-management | Self-care behavior score |  | 14.9 (4.64) | 23.9 (6.49) | - | - | - | 0.001 |  |
| Cheng (2019) [62] | QOL | Adjusted model QOL | 18 weeks |  |  | 4.151 | 1.291 | 7.012 | **0.004** | 0.21 |
| Yucel (2015) [63] | QOL | SF-36-physical health | 12 weeks | 40.0 (3.0) | 41.0 (4.0) | - | - | - |  |  |
|  |  | SF-36-mental health |  | 29.0 (5.0) | 35.0 (3.0) | - | - | - |  |  |
|  | Glycemic | FBG |  | 140.0 (31.0) | 139.5 (32.0) | - | - | - |  |  |
| Sreedevi (2017) [39] | QOL in Yoga group | Physical | 12 weeks |  |  | -2.47 (14.3) | - | - | 0.34 |  |
|  |  | Psychological |  |  |  | 0.39 (16.34) | - | - | 0.89 |  |
|  |  | Social |  |  |  | -1.16 (20.52) | - | - | 0.75 |  |
|  |  | Environmental |  |  |  | 4.45 (12.78) | - | - | 0.06 |  |
|  | QOL in Peer support group | Physical |  |  |  | 3.98 (19.2) | - | - | 0.3 |  |
|  |  | Psychological |  |  |  | 0.0007 (17.9) | - | - | 1 |  |
|  |  | Social |  |  |  | 7.69 (15.03) | - | - | **0.015** |  |
|  |  | Environmental |  |  |  | 4.07 (8.76) | - | - | **0.026** |  |
| Singh (2020) [40] | QOL | DQOL | 14 weeks | 46.9 (5.4) | 70.7 (7.4) | - | - | - | **<0.05** |  |
|  |  | HbA1c |  | 8.6 (0.6) | 8.1 (0.7) | - | - | - | <0.05 |  |
| Umphonsathien (2022) [64] | QOL in 2 day/week VLCD | SF-36 (point) | 18 weeks | 2444 (151) | 2757 (118) | - | - | - | 0.055 |  |
|  |  | BMI |  | 29.9 (1.6) | 27.8 (1.5) | - | - | - | 0.001 |  |
|  |  | FPG |  | 156.0 (13.0) | 130.9 (9.9) | - | - | - | 0.051 |  |
|  | QOL in 4 day/week VLCD | SF-36 (point) |  | 2081 (151) | 2697 (118) | - | - | - | **<0.001** |  |
|  |  | BMI |  | 31.0 (1.6) | 27.4 (1.5) | - | - | - | <0.001 |  |
|  |  | FPG |  | 159.6 (12.8) | 119.9 (9.9) | - | - | - | 0.003 |  |
| Chaveepojnkamjorn (2009) [65] | QOL | Total QOL | 24 weeks | 80.6 (7.5) | 96.2 (5.8) | - | - | - | **<0.001** |  |
|  |  | Physical health |  | 23.4 (2.9) | 27.9 (2.8) | - | - | - | <0.001 |  |
|  |  | Psychological |  | 21.1 (2.7) | 25.2 (2.5) | - | - | - | <0.001 |  |
|  |  | Social relationships |  | 10.1 (1.3) | 12.2 (1.6) | - | - | - | <0.001 |  |
|  |  | Environment |  | 25.9 (3.2) | 30.9 (3.4) | - | - | - | <0.001 |  |
| Saghaee (2020) [41] | QOL | Overall QOL score | 4 weeks | 54.41 (7.73) | 58.82 (7.71) | - | - | - |  |  |
|  | Self-care activity | Overall score |  | 58.19 (11.07) | 64.44 (13.81) | - | - | - |  |  |
| Torabizadeh (2018) [42] | QOL | Overall | 19 weeks | 117.5 (18.7) | 138.0 (15.2) | - | - | - | **<0.001** |  |
|  |  | Role limitation due to physical health |  | 23.4 (4.4) | 24.3 (4.2) | - | - | - | 0.11 |  |
|  |  | Physical endurance |  | 22.2 (5.6) | 24.0 (5.1) | - | - | - | <0.001 |  |
|  |  | General Health |  | 8.3 (2.5) | 12.6 (2.0) | - | - | - | <0.001 |  |
|  |  | Treatment satisfaction |  | 14.1 (3.7) | 16.4 (3.4) | - | - | - | <0.001 |  |
|  |  | Symptom suffering |  | 10.1 (3.3) | 11.8 (2.4) | - | - | - | <0.001 |  |
|  |  | Financial Worries |  | 13.2 (4.5) | 15.2 (3.8) | - | - | - | <0.001 |  |
|  |  | Emotional/mental health |  | 16.0 (4.2) | 21.6 (3.2) | - | - | - | <0.001 |  |
|  |  | Diet advise tolerance |  | 10.4 (2.8) | 11.5 (2.9) | - | - | - | 0.008 |  |
|  | Self-care behavior | Healthy eating |  | 51.5 (28.4) | 54.2 (30.4) | - | - | - | 0.33 |  |
|  |  | Engaging in physical activity |  | 68.0 (34.6) | 76.9 (29.8) | - | - | - | 0.01 |  |
|  | Glycemic | HbA1c |  | 8.9 (1.9) | 8.2 (1.6) | - | - | - | <0.001 |  |
| Sekhar (2019) [43] | QOL | PCS | 13 weeks | 25.6 (7.3) | 42.9 (9.7) | - | - | - | **<0.005** |  |
|  |  | MCS |  | 28.8 (7.1) | 48.8 (8.4) | - | - | - | <0.005 |  |
|  |  | Physical functioning |  | 17.6 (5.3) | 31.5 (7.2) | - | - | - | <0.005 |  |
|  |  | Role limitation due to physical health |  | 9.5 (4.6) | 32.6 (8.5) | - | - | - | <0.005 |  |
|  |  | Role limitation due to emotional health |  | 9.1 (3.2) | 23.2 (3.6) | - | - | - | <0.005 |  |
|  |  | Energy/fatigue |  | 36.5 (7.6) | 56.3 (9.8) | - | - | - | <0.005 |  |
|  |  | Emotional well-being |  | 27.3 (8.8) | 56.2 (9.5) | - | - | - | <0.005 |  |
|  |  | Social functioning |  | 42.4 (9.4) | 59.4 (7.7) | - | - | - | <0.005 |  |
|  |  | Bodily pain |  | 39.3 (7.6) | 52.4 (10.6) | - | - | - | <0.005 |  |
|  |  | General health |  | 35.8 (9.7) | 55.2 (8.4) | - | - | - | <0.005 |  |
| Arora (2009) [44] | Well-being in PRT group | General well-being score | 8 weeks | 44.0 (5.0) | 48.0 (6.0) | - | - | - | **<0.05** |  |
|  | Well-being in AE group | General well-being score |  | 41.0 (5.0) | 42.0 (4.0) | - | - | - |  |  |
|  | HbA1c in PRT group | HbA1c |  | 7.6 (1.4) | 6.2 (0.8) | - | - | - | <0.05 |  |
|  | HbA1c in AE group | HbA1c |  | 8.1 (0.9) | 6.7 (0.9) | - | - | - |  |  |
|  | Anthropometric, PRT group | BMI |  | 27.0 (4.1) | 26.8 (4.1) | - | - | - |  |  |
|  | Anthropometric, AE group | BMI |  | 26.2 (3.2) | 25.8 (3.8) | - | - | - |  |  |
| Shahsavari (2021) [45] | QOL | QOL | 13 weeks | 27.1 (2.45) | 41.3 (2.7) | - | - | - | **<0.001** |  |
| Ebrahimi (2018) [10] | QOL | Total | 12 weeks |  | 71.03 (21.88) | - | - | - | **0.001** |  |
|  |  | Physical |  |  | 14.86 (7.34) | - | - | - | 0.001 |  |
|  |  | Mental |  |  | 16.15 (5.83) | - | - | - | 0.001 |  |
|  |  | Social |  |  | 9.74 (4.69) | - | - | - | 0.001 |  |
|  |  | Economic |  |  | 3.36 (3.69) | - | - | - | 0.013 |  |
|  |  | Disease and treatment |  |  | 24.31 (10.48) | - | - | - | 0.001 |  |
| Rasoul (2019) [11] | QOL | Mean QOL score | 20 weeks | 37.8 (3.6) | 59.1 (2.2) | - | - | - |  |  |
|  |  | BMI |  | 29.8 (3.6) | 27.3 (3.5) | - | - | - |  |  |
|  |  | FBG |  | 250.3 (50.5) | 131.1 (16.0) | - | - | - |  |  |
| Browning (2016) [66] | QOL | Physical domain | 52 weeks | 62.6 (12.9) | 60.4 (12.2) | - | - | - |  |  |
|  |  | Psychological domain |  | 68.8 (14.5) | 62.6 (15.3) | - | - | - |  |  |
|  |  | Social relationship domain |  | 64.2 (13.9) | 63.4 (13.6) | - | - | - |  |  |
|  |  | Environment domain |  | 65.4 (14.5) | 63.5 (14.7) | - | - | - |  |  |
|  | Summary of diabetes self-care activities (SDSCA) | General diet |  | 5.4 (1.8) | 5.3 (1.5) | - | - | - |  |  |
|  |  | Specific diet |  | 4.1 (1.5) | 4.4 (1.2) | - | - | - |  |  |
|  |  | Exercise |  | 5.3 (2.1) | 5.1 (1.7) | - | - | - |  |  |
|  |  | Blood glucose monitoring |  | 1.5 (1.8) | 2.5 (2.0) | - | - | - |  |  |
|  |  | Foot care |  | 4.5 (2.8) | 4.9 (2.3) | - | - | - |  |  |
|  | Glycemic | HbA1c |  | 10.6 (2.1) | 6.7 (1.7) | - | - | - |  |  |
|  | Anthropometric | BMI |  | 26.2 (3.7) | 26.2 (3.7) | - | - | - |  |  |
|  |  | Body weight |  | 70.1 (11.7) | 70.1 (11.7) | - | - | - |  |  |
| Jamshidpour (2020) [46] | QOL | General health | 8 weeks | 54.6 (15.8) | 62.6 (26.8) | - | - | - | 0.2 |  |
|  |  | Physical function |  | 67.1 (17.8) | 66.8 (23.6) | - | - | - | 0.93 |  |
|  |  | Role limitation due to physical problems |  | 79.8 (22.6) | 81.4 (38.0) | - | - | - | 0.89 |  |
|  |  | Role limitation due to emotional problems |  | 80.9 (21.6) | 85.5 (28.9) | - | - | - | 0.63 |  |
|  |  | Social function |  | 86.6 (31.6) | 78.6 (27.4) | - | - | - | 0.43 |  |
|  |  | Bodily pain |  | 74.3 (28.5) | 75.9 (24.5) | - | - | - | 0.87 |  |
|  |  | Energy and fatigue |  | 63.6 (21.2) | 58.6 (26.2) | - | - | - | 0.46 |  |
|  |  | Emotional well-being |  | 80.0 (22.2) | 76.3 (24.1) | - | - | - | 0.52 |  |
|  |  | Physical health |  | 68.9 (11.7) | 71.7 (23.7) | - | - | - | 0.63 |  |
|  |  | Mental health |  | 77.8 (21.0) | 74.4 (23.4) | - | - | - | 0.64 |  |
| Abraham (2020) [47] | QOL | RMANOVA QOL total score | 22 weeks | 2.85 (0.31) | 2.72 (0.3) | - | - | - |  | 0.62 |
|  | Summary of diabetes self-care activities schedule (SDSCA) RMANOVA | SDSCA-General diet |  | 3.9 (2.0) | 4.9 (1.6) | - | - | - |  | 0.96 |
|  |  | SDSCA-specific diet |  | 4.5 (1.6) | 5.4 (1.3) | - | - | - |  | 0.93 |
|  |  | SDSCA-exercise |  | 2.3 (2.3) | 3.8 (2.1) | - | - | - |  | 1.27 |
|  |  | SDSCA-glucose testing |  | 1.0 (0.6) | 1.1 (0.5) | - | - | - |  | 0.36 |
|  |  | SDSCA-foot care |  | 0.5 (0.6) | 0.4 (0.7) | - | - | - |  | 0.09 |
|  | Glycemic | HbA1c |  | 8.6 (0.8) | 8.1 (0.8) | - | - | - |  | 1.06 |
| Akinci (2018) [67] | QOL group 1 | EQ-5D score | 8 weeks | 0.46 (0.33) | 0.72 (0.24) | - | 0.14 | 0.36 |  |  |
|  |  | HbA1c |  | 8.01 (1.23) | 7.20 (0.93) | - | -1.24 | -0.36 |  |  |
|  |  | BMI |  | 31.81 (6.36) | 30.75 (5.84) | - | -1.62 | -0.48 |  |  |
|  |  | FBG |  | 170.04 (61.47) | 130.59 (41.0) | - | -5.38 | 0.84 |  |  |
|  | QOL group 2 | EQ-5D score |  | 0.61 (0.2) | 0.77 (0.08) | - | 0.07 | 0.23 |  |  |
|  |  | HbA1c |  | 8.50 (1.48) | 7.59 (0.99) | - | -1.45 | -0.36 |  |  |
|  |  | BMI |  | 32.48 (4.43) | 31.83 (2.85) | - | -2.17 | 0.87 |  |  |
|  |  | FBG |  | 183.14 (59.89) | 147.73 (32.0) | - | -57.29 | -13.51 |  |  |
| Rias (2020) [48] | QOL | SF-36 Physical component score (Walking group) | 8 weeks | 45.16 (6.34) | 60.81 (3.24) | - | - | - | <0.001 |  |
|  |  | SF-36 Mental component score (Mental group) |  | 47.50 (9.01) | 61.37 (12.53) | - | - | - | <0.001 |  |
|  |  | SF-36 Total QOL (Walking group) |  | 46.33 (6.63) | 61.09 (7.15) | - | - | - | **<0.001** |  |
|  |  | FBG |  | 303.95 (67.95) | 288.00 (67.70) | - | - | - | <0.001 |  |
| Peimani (2018) [49] | QOL | QOL score | 26 weeks | 150.34 (20.49) | 198.75 (26.50) | - | - | - | **<0.001** |  |
|  |  | HbA1c |  | 7.29 (1.33) | 6.98 (1.31) | - | - | - | 0.045 |  |
|  |  | BMI |  | 28.11 (5.29) | 27.52 (5.52) | - | - | - | 0.162 |  |
|  |  | Diabetes self-management score |  | 28.11 (10.26) | 37.35 (10.54) | - | - | - | <0.001 |  |
| Tapehsari (2020) [50] | QOL | Physical domain | 12 weeks | 23.78 (3.17) | 27.42 (3.34) | - | - | - |  |  |
|  |  | Psychological domain |  | 17.76 (2.72) | 21.44 (3.24) | - | - | - |  |  |
|  |  | Social relationship domain |  | 8.70 (2.10) | 9.46 (2.70) | - | - | - |  |  |
|  |  | Environment domain |  | 24.24 (3.15) | 27.02 (4.68) | - | - | - |  |  |
|  |  | FBG |  | 152.55 (17.68) | 136.63 (16.86) | - | - | - |  |  |
| Jaipakdee (2015) [68] | QOL | QOL score | 26 weeks | 58.4 (6.2) | 64.1 (5.8) | - | - | - | **<0.001** |  |
|  |  | HbA1c |  | 8.2 (1.5) | 7.8 (1.4) | - | - | - | 0.334 |  |
|  |  | Health behavior score |  | 124.4 (12.5) | 135.9 (10.5) | - | - | - | <0.001 |  |
|  |  | Body weight |  | 66.6 (12.2) | 64.7 (12.7) | - | - | - | 0.001 |  |
| Wongrochananan (2015) [69] | QOL | DQOL | 26 weeks | 52.55 (9.28) | 57.87 (6.87) | - | - | - |  |  |
|  |  | Summary of Diabetes Self-care Activities Measure (SDSCA) |  | 63.57 (17.90) | 73.03 (16.02) | - | - | - | p<0.01 |  |
|  |  | BMI |  | 26.75 (5.00) | 26.74 (5.04) | - | - | - |  |  |
|  |  | HbA1c |  | 7.74 (1.66) | 7.46 (1.67) | - | - | - |  |  |
| Anderson (2009) [51] | QOL | PAID QOL score | 104 weeks | 29.3 (19.8) | 20.2 (18.9) | - | - | - | **p<0.001** |  |
|  |  | HbA1c |  | 7.57 (1.94) | 7.62 (1.80) | - | - | - |  |  |
| Dede (2015) [75] | QOL | Physical functioning | 12 weeks | 85.0 (18.0) | 90.0 (20.0) | - | - | - | **0.02** |  |
|  |  | Physical problems |  | 75.0 (50.0) | 100.0 (25.0) | - | - | - | 0.367 |  |
|  |  | Pain |  | 80.0 (39.0) | 74.0 (38.0) | - | - | - | 0.936 |  |
|  |  | General health perception |  | 60.0 (27.0) | 62.0 (34.0) | - | - | - | 0.267 |  |
|  |  | Vitality |  | 65.0 (30.0) | 70.0 (28.0) | - | - | - | 0.216 |  |
|  |  | Social functioning |  | 87.5 (25.0) | 87.5 (25.0) | - | - | - | 0.776 |  |
|  |  | Emotional problems |  | 66.6 (67.0) | 66.6 (67.0) | - | - | - | 0.346 |  |
|  |  | Mental health |  | 68.0 (18.0) | 72.0 (20.0) | - | - | - | **0.023** |  |
|  |  | HbA1c |  | 7.0 (0.8) | 6.7 (1.0) | - | - | - | 0.079 |  |
|  |  | BMI |  | 30.9 (4.6) | 30.0 (4.5) | - | - | - | 0.289 |  |
| Cani (2015) [70] | QOL | DQOL score | 26 weeks | 157.21 (13.28) | 152.06 (14.98) | - | - | - | **<0.001** |  |
|  | Medication adherence | Adherence Morisky-Green (%) |  | 17.6 | 70.6 | - | - | - | <0.001 |  |
|  |  | HbA1c |  | 9.78 (1.55) | 9.21 (1.41) | - | - | - | <0.001 |  |
| Nouripour (2021) [52] | QOL score in high-carbohydrate group | Physical composite score | 10 weeks | 262.4 (66.4) | 290.4 (66.7) | - | - | - | **0.001** |  |
|  |  | Physical functioning |  | 74.5 (20.3) | 79.4 (21.1) | - | - | - | 0.051 |  |
|  |  | Role-physical |  | 66.9 (22.1) | 71.0 (17.9) | - | - | - | 0.31 |  |
|  |  | Pain |  | 60.3 (17.4) | 69.5 (17.8) | - | - | - | <0.001 |  |
|  |  | General health perception |  | 60.7 (26.1) | 70.6 (24.0) | - | - | - | 0.039 |  |
|  |  | Mental composite score |  | 273.9 (71.1) | 288.2 (59.3) | - | - | - | 0.089 |  |
|  |  | Vitality |  | 60.1 (13.2) | 64.5 (14.6) | - | - | - | 0.036 |  |
|  |  | Social functioning |  | 69.8 (23.9) | 80.2 (18.2) | - | - | - | 0.025 |  |
|  |  | Role-emotional |  | 73.9 (18.8) | 73.9 (18.2) | - | - | - | 0.99 |  |
|  |  | Mental health |  | 70.2 (15.1) | 71.1 (14.1) | - | - | - | 0.73 |  |
|  | QOL score in high-protein group | Physical composite score |  | 278.8 (66.1) | 285.8 (67.4) | - | - | - | 0.33 |  |
|  |  | Physical functioning |  | 77.6 (18.9) | 78.1 (17.8) | - | - | - | 0.38 |  |
|  |  | Role-physical |  | 70.7 (22.8) | 68.5 (20.6) | - | - | - | 0.77 |  |
|  |  | Pain |  | 61.6 (19.5) | 66.7 (19.0) | - | - | - | 0.16 |  |
|  |  | General health perception |  | 68.9 (24.4) | 72.5 (22.1) | - | - | - | 0.29 |  |
|  |  | Mental composite score |  | 277.5 (65.5) | 279.1 (71.1) | - | - | - | 0.71 |  |
|  |  | Vitality |  | 61.4 (20.1) | 62.5 (19.8) | - | - | - | 0.86 |  |
|  |  | Social functioning |  | 72.4 (27.0) | 75.4 (21.0) | - | - | - | 0.34 |  |
|  |  | Role-emotional |  | 74.4 (21.2) | 71.0 (24.2) | - | - | - | 0.009 |  |
|  |  | Mental health |  | 65.7 (17.1) | 70.2 (19.2) | - | - | - | 0.045 |  |
| Sunil (2020) [53] | QOL median score | Physical health | 26 weeks | 56 | 63 | - | - | - | **<0.001** |  |
|  |  | Psychological |  | 50 | 63 | - | - | - | <0.001 |  |
|  |  | Social Relationships |  | 56 | 69 | - | - | - | <0.001 |  |
|  |  | Environmental |  | 56 | 63 | - | - | - | <0.001 |  |
| Shi (2018) [71] | QOL (Baseline to 6 month) | Physical composite score | 52 weeks |  |  | -9.03 | - | - | <0.05 |  |
|  |  | Mental composite score |  |  |  | -11.9 | - | - | <0.05 |  |
|  |  | SF-36 |  |  |  | -10.99 | - | - | **<0.05** |  |
|  |  | BMI |  |  |  | 1.32 | - | - |  |  |
|  | QOL (6 to 12 month) | Physical composite score |  |  |  | -10.36 | - | - | <0.05 |  |
|  |  | Mental composite score |  |  |  | -9.24 | - | - | <0.05 |  |
|  |  | SF-36 |  |  |  | -14.43 | - | - | <0.05 |  |
|  |  | BMI |  |  |  | 1.57 | - | - |  |  |
| Kong (2019) [72] | QOL | Physical functioning | 39 weeks | 67.90 (22.2) | 76.45 (24.4) | - | - | - | <0.001 |  |
|  |  | Role-physical |  | 75.00 (38.8) | 91.2 (26.4) | - | - | - | <0.001 |  |
|  |  | Pain |  | 78.61 (20.1) | 78.44 (19.5) | - | - | - | 0.92 |  |
|  |  | General health perception |  | 44.84 (17.1) | 42.87 (15.8) | - | - | - | 0.17 |  |
|  |  | Vitality |  | 46.26 (14.48) | 42.17 (12.42) | - | - | - | 0.002 |  |
|  |  | Social functioning |  | 85.63 (20.25) | 84.79 (19.83) | - | - | - | 0.57 |  |
|  |  | Role-emotional |  | 85.82 (33.55) | 96.27 (19.02) | - | - | - | <0.001 |  |
|  |  | Mental health |  | 59.41 (20.17) | 59.20 (16.87) | - | - | - | 0.9 |  |
|  |  | Physical composite score |  | 48.34 (12.62) | 52.31 (8.41) | - | - | - | **<0.001** |  |
|  |  | Mental composite score |  | 43.86 (8.46) | 40.98 (7.34) | - | - | - | <0.001 |  |
|  |  | BMI |  | 24.35(3.15) | 23.14 (3.26) | - | - | - | 0.14 |  |
|  |  | SBP |  | 128.99 (11.06) | 129.05 (8.97) | - | - | - | 0.95 |  |
|  |  | DBP |  | 75.06 (7.21) | 73.20 (5.94) | - | - | - | 0.006 |  |
| Safavi (2011) [73] | QOL | QOL score | 10 weeks | 231.1 (4.5) | 279.2 (5.1) | - | - | - | **0** |  |
| Nazir (2020) [54] | QOL | QOL EQ5D score | 13 weeks | 0.47 (0.62) | 0.61 (0.69) | - | - | - | **<0.001** |  |
|  |  | EQ-VAS score |  | 65.33 (70.00) | 68.37 (70.0) | - | - | - | <0.001 |  |
|  |  | HbA1c |  | 9.45 (9.00) | 8.98 (8.70) | - | - | - | <0.001 |  |
| Yang (2022) [74] | QOL | Role emotional | 52 weeks | 59.29 (26.64) | 68.51 (22.02) | - | - | - | **0** |  |
|  |  | Vitality |  | 68.72 (10.03) | 72.55 (10.42) | - | - | - | 0 |  |
|  |  | Social functioning |  | 92.39 (13.21) | 95.15 (11.39) | - | - | - | 0 |  |
|  |  | Mental health |  | 68.68 (9.34) | 74.6 (8.63) | - | - | - | 0 |  |
|  |  | Physical functioning |  | 69.04 (15.66) | 85.43 (16.25) | - | - | - | 0 |  |
|  |  | Role physical |  | 42.95 (7.33) | 62.34 (11.83) | - | - | - | 0 |  |
|  |  | Bodily pain |  | 70.19 (16.81) | 74.87 (15.32) | - | - | - | 0 |  |
|  |  | General health |  | 66.10 (15.91) | 71.38 (14.68) | - | - | - | 0 |  |
|  |  | HbA1c |  | 7.44 (1.76) | 6.18 (0.73) | - | - | - | 0 |  |
|  |  | BMI |  | 24.94 (2.96) | 23.66 (2.86) | - | - | - | 0 |  |
| Arovah (2018) [55] | QOL | EQ-VAS score | 24 weeks | 77 | 83 | - | - | - |  |  |
|  |  | Mobility |  | 1.14 | 1.2 | - | - | - |  |  |
|  |  | Self-care |  | 1 | 1.05 | - | - | - |  |  |
|  |  | Daily activity |  | 1.1 | 1.05 | - | - | - |  |  |
|  |  | Pain |  | 1.24 | 1.25 | - | - | - |  |  |
|  |  | Anxiety |  | 1.19 | 1.15 | - | - | - |  |  |
|  | Physical activity | Daily steps |  | 4876 | 8214 | - | - | - |  |  |
|  |  | Walking (min/week) |  | 34 | 162 | - | - | - |  |  |
|  |  | Moderate-to-vigorous-intensity physical activity(min/week) |  | 102 | 219 | - | - | - |  |  |
|  | Glycemic | HbA1c |  | 7.85 | 7.24 | - | - | - |  |  |

QOL: Quality of life, BMI: Body mass index, HbA1c: Glycated haemoglobin, FBG: Fasting blood glucose, PCS: Physical component summary, MCS: Mental component summary, DQOL: Diabetes Quality of life, EQ5D: European Quality of life 5D, EQ-VAS: EuroQol-Visual analogue scale, PRT: Progressive resistance training, AE: Aerobic exercise, PAID: Problem areas in diabetes
