# Supplementary material for: The effect of health behavior interventions to manage Type 2 diabetes on the quality of life in low-and middle-income countries: A systematic review and meta-analysis
Source: PLoS One. 2023 Oct 16;18(10):e0293028. doi: 10.1371/journal.pone.0293028 (PMC10578590; doi:10.1371/journal.pone.0293028)
Supplement: S4 Table — (DOCX) [file pone.0293028.s009.docx]

**S4 Table. Quality assessment of studies using Joanna Briggs Institute (JBI) Critical Appraisal Checklist for Randomized Controlled Trials**

| **Author (Year)** | **ALLOCATION CONCEALMENT for All outcomes** | **APPROPRIATE STATISTICAL ANALYSIS for All outcomes** | **APPROPRIATE TRIAL DESIGN for All outcomes** | **BLINDING OF OUTCOMES ASSESSORS for All outcomes** | **BLINDING OF PARTICIPANTS TO TREATMENT ASSIGNMENT for All outcomes** | **BLINDING OF PERSONNEL TO TREATMENT ASSIGNMENT for All outcomes** | **COMPLETE FOLY UP for All outcomes** | **IDENTICAL TREATMENT OF TREATMENT GROUPS OTHER THAN THE INTERVENTION OF INTEREST for All outcomes** | **INTENTION-TO-TREAT ANALYSIS for All outcomes** | **RELIABLE MEASUREMENT OF OUTCOMES for All outcomes** | **SAME OUTCOME MEASUREMENT FOR TREATMENT GROUPS for All outcomes** | **TREATMENT GROUPS SIMILAR AT THE BASELINE for All outcomes** | **True randomization for All outcomes** | **Quality** |
| --- | --- | --- | --- | --- | --- | --- | --- | --- | --- | --- | --- | --- | --- | --- |
| Browning (2016) | Y | Y | Y | N | N | N | Y | Y | Y | Y | Y | Y | Y | Good |
| Castillo-Hernandez (2021) | Y | Y | Y | Y | N | N | Y | Y | Y | Y | Y | Y | Y | Good |
| Chaveepojnkamjorn (2009) | Y | Y | Y | N | unclear | Y | unclear | Y | N | Y | Y | Y | Y | Good |
| Ebrahimi (2018) | unclear | Y | Y | Y | N | unclear | Y | Y | N | Y | Y | Y | Y | Good |
| Jaipakdee (2015) | unclear | Y | Y | N | N | N | Y | Y | N | Y | Y | Y | Y | Good |
| Jamshidpour (2020) | unclear | Y | Y | N | N | Y | Y | Y | unclear | Y | Y | Y | Y | Good |
| Mash (2014) | unclear | Y | Y | N | N | N | Y | Y | Y | unclear | Y | Y | Y | Good |
| Peimani (2018) | unclear | Y | Y | N | N | N | Y | Y | unclear | Y | Y | Y | Y | Good |
| Rasoul (2019) | unclear | Y | Y | N | N | N | Y | Y | unclear | unclear | Y | Y | Y | Fair |
| Umphonsathien (2022) | unclear | Y | Y | N | N | N | Y | Y | Y | unclear | Y | Y | Y | Good |
| Abraham (2020) | N | Y | Y | N | N | N | Y | Y | Y | N | Y | Y | Y | Good |
| Butt (2016) | Y | Y | Y | N | N | N | Y | Y | N | N | Y | Y | Y | Good |
| Maharaj (2015) | unclear | Y | Y | N | N | N | unclear | Y | N | unclear | Y | Y | Y | Fair |
| Rias (2020) | Y | Y | Y | Y | N | Y | Y | Y | Y | Y | Y | Y | Y | Good |
| Rondhianto (2018) | unclear | Y | Y | N | N | N | unclear | Y | N | unclear | Y | Y | Y | Fair |
| Shahsavari (2021) | Y | Y | Y | Y | unclear | N | Y | Y | unclear | Y | Y | Y | Y | Good |
| Sreedevi (2017) | Y | Y | Y | N | N | N | Y | Y | unclear | Y | Y | Y | Y | Good |
| Tapehsari (2020) | Y | Y | Y | N | N | N | Y | Y | N | Y | Y | Y | Y | Good |
| Torabizadeh (2018) | unclear | Y | Y | Y | N | N | Y | Y | unclear | Y | Y | Y | Y | Good |
| Wongrochananan (2015) | unclear | Y | Y | N | N | N | Y | Y | Y | Y | Y | Y | Y | Good |
| Akinci (2018) | Y | Y | Y | Y | Y | unclear | Y | Y | Y | N | Y | Y | Y | Good |
| Cheng (2021) | Y | Y | Y | Y | N | N | Y | Y | Y | Y | Y | Y | Y | Good |
| Sekhar (2019) | Y | Y | Y | N | N | N | N | Y | N | Y | Y | Y | Y | Good |
| Singh (2020) | Y | Y | Y | Y | N | N | Y | Y | unclear | unclear | Y | Y | Y | Good |
| Yucel (2018) | unclear | Y | Y | N | N | N | Y | Y | N | Y | Y | Y | Y | Good |
| Arora (2009) | unclear | Y | Y | N | N | N | Y | Y | N | N | Y | Y | Y | Fair |
| Azami (2018) | Y | Y | Y | Y | unclear | Y | Y | Y | Y | Y | Y | Y | Y | Good |
| Kong (2019) | unclear | Y | Y | Y | N | N | N | Y | N | N | Y | N | Y | Fair |
| Mohammadi (2018) | unclear | Y | Y | N | N | N | unclear | Y | N | Y | Y | Y | Y | Fair |
| Nazir (2020) | unclear | Y | Y | N | N | N | Y | Y | N | N | Y | Y | Y | Fair |
| Safavi (2011) | unclear | Y | Y | N | N | N | N | Y | N | Y | Y | Y | Y | Fair |
| Shenoy (2010) | unclear | Y | Y | N | N | N | Y | Y | unclear | N | Y | Y | Y | Fair |
| Wichit (2017) | Y | Y | Y | N | N | unclear | Y | Y | Y | unclear | Y | Y | Y | Good |
| Yang (2022) | unclear | Y | Y | N | N | N | Y | Y | Y | N | Y | Y | Y | Good |
| Zuo (2020) | Y | Y | Y | Y | N | N | Y | Y | N | Y | Y | Y | Y | Good |
| Lyu (2021) | Y | Y | Y | N | N | N | Y | Y | Y | Y | Y | Y | Y | Good |
| Anderson (2009) | Y | Y | Y | N | N | N | Y | Y | N | N | Y | Y | Y | Good |
| Cani (2015) | unclear | Y | Y | N | N | N | Y | Y | N | N | Y | Y | Y | Fair |
| Dede (2015) | unclear | Y | Y | N | N | N | Y | Y | N | N | Y | Y | Y | Fair |
| SunilKumar (2020) | Y | Y | Y | N | N | N | Y | Y | unclear | N | Y | Y | Y | Good |
| Shi (2018) | unclear | Y | Y | N | N | Y | Y | Y | Y | N | Y | Y | Y | Good |
| Nouripour (2021) | unclear | Y | Y | N | Y | N | Y | Y | Y | N | Y | Y | Y | Good |
| Wattana (2007) | Y | Y | Y | N | N | N | Y | Y | Y | Y | Y | Y | Y | Good |
| Saghaee (2020) | N | Y | Y | Y | unclear | N | Y | Y | unclear | N | Y | Y | Y | Good |
| Arovah (2018) | N | Y | Y | Y | N | N | Y | Y | Y | N | Y | Y | Y | Good |

Y = Yes = 1, No = No = 0, Unclear = 0

Summary scores were obtained by adding item-specific scores for each studies; quality rating was given based on summary scores, i.e., ≥8 score = good, 6-7 = fair, and ≤5 = poor quality.
