# Supplementary material for: The effect of health behavior interventions to manage Type 2 diabetes on the quality of life in low-and middle-income countries: A systematic review and meta-analysis
Source: PLoS One. 2023 Oct 16;18(10):e0293028. doi: 10.1371/journal.pone.0293028 (PMC10578590; doi:10.1371/journal.pone.0293028)
Supplement: S5 Table — (DOCX) [file pone.0293028.s010.docx]

**S5 Table. GRADE certainty of evidence**

| Outcomes | SMD, 95% CI, I^2^ | Number of participants (studies) | Risk of Bias | Inconsistency | Indirectness | Imprecision | Publication Bias | GRADE Quality of Evidence |
| --- | --- | --- | --- | --- | --- | --- | --- | --- |
| Mean Quality of life | SMD: 1.62, 95% CI: 0.65 to 2.60, I^2^: 96% | 3867 (25) | Not serious; not downgraded^ⱡ^ | Serious* | Not serious; not downgraded | Serious^¥^ | Serious* | ⊙⊙⊙○ Moderate quality |
| PCS | SMD: 0.76, 95% CI: -0.03 to 1.56 I^2^: 94% | 834 (7) | Not serious; not downgraded^ⱡ^ | Serious* | Not serious; not downgraded | Serious* | Not downgraded^^^ | ⊙⊙○○ Low quality |
| MCS | SMD: 0.43, 95% CI: -0.30 to 1.16 I^2^: 94% | 834 (7) | Not serious; not downgraded^ⱡ^ | Serious* | Not serious; not downgraded | Serious* | Not downgraded^^^ | ⊙⊙○○  Low quality |

^ⱡ^Majority of the trials scored > 6 on the JBI critical appraisal checklist for RCTs, hence not downgraded
*Downgraded by one level
^¥^SMD ≥ 0.8, hence certainty was upgraded by one place

^^^Funnel plots not assessed for publication bias due to less than 10 studies in the meta-analysis

High quality: We are very confident that the true effect lies close to that of the estimate of the effect
Moderate quality: We are moderately confident in the effect estimate; the true effect is likely to be close to the estimate of the effect, but there is a possibility that it is substantially different
Low quality: Our confidence in the effect estimate is limited; the true effect may be substantially different from the estimate of the effect
Very low quality: We have very little confidence in the effect estimate; the true effect is likely to be substantially different from the estimate of effect
