# Supplementary material for: The effect of health behavior interventions to manage Type 2 diabetes on the quality of life in low-and middle-income countries: A systematic review and meta-analysis
Source: PLoS One. 2023 Oct 16;18(10):e0293028. doi: 10.1371/journal.pone.0293028 (PMC10578590; doi:10.1371/journal.pone.0293028)
Supplement: S6 Table — (DOCX) [file pone.0293028.s011.docx]

**S6 Table. Subgroup analyses**

| **Subgroups** | **Mean QOL** | **PCS** | **MCS** |
| --- | --- | --- | --- |
|  | SMD (95% CI), *I*^2^, (n) | SMD (95% CI), *I*^2^, (n) | SMD (95% CI), *I*^2^, (n) |
| **Intervention Type** |  |  |  |
| Self-management education | 1.69 (0.49, 2.90), 96.6, (n=22) | 0.89 (-0.36, 2.14), 97.0, (n=3) | 0.84 (-1.02, 2.69), 98.7, (n=3) |
| Structured diet and/or exercise | 1.39 (0.54, 2.24), 93.4, (n=5) | 0.70 (-0.47, 1.86), 89.9, (n=5) | 0.16 (-0.33, 0.64), 69.8, (n=5) |
| Test for subgroup difference | Q=0.17, p-value=0.63 | Q=0.05, p-value=0.82 | Q=0.40, p-value=0.48 |
| **Intervention Setting** |  |  |  |
| Hospital/diabetes clinic | 1.74 (0.49, 3.01), 97.0, (n=21) | 0.51 (-0.18, 1.19), 92.8, (n=6) | 0.49 (-0.38, 1.36), 94.9, (n=6) |
| Community health centre | 1.23 (0.59, 1.86), 94.1, (n=4) | 3.22 (2.26, 4.17), (n=1) | 1.23 (0.56, 1.90), (n=1) |
| Home/web-based | 1.35 (-0.41, 3.13), 94.1, (n=2) | 0.17 (-0.06, 0.39), (n=1) | -0.64 (-0.87, -0.41), (n=1) |
| Test for subgroup difference | Q=0.49, p-value=0.78 | Q=37.52, p-value <0.001 | Q=30.60, p-value <0.001 |
| **Intervention Duration** |  |  |  |
| <12 weeks | 3.60 (-1.51 to 8.73), 98.1 (n=5) | 0.21 (-0.43 to 0.85) 77.0 (n=4) | 0.67 (-0.31 to 1.66) 95.8 (n=4) |
| ≥ 12 weeks | 1.20 (0.66 to 1.74), 96.0% (n=22) | 0.63 (-0.76, 2.00), 98.1 (n=4) | 0.88 (-0.58 to 2.34) 92.0 (n=4) |
| Test for subgroup difference | Q=0.84, p-value=0.36 | Q=0.29, p-value=0.58 | Q=0.05, p-value =0.814 |
| **Methodology Quality** |  |  |  |
| Good | 1.25 (0.66 to 1.84), 96.1% (n=21) | 0.85 (-0.05 to 1.76), 93.8 (n=7) | 0.59 (-0.17 to 1.35),94.1% (n=7) |
| Fair | 3.03 (-1.22 to 7.25), 97.8% (n=6) | 0.16 (-0.06 to 0.39), (n=1) | -0.63 (-0.87 to -0.41), (n=1) |
| Test for subgroup difference | Q=0.66, p-value=0.41 | Q=2.09, p-value=0.14 | Q=9.08, p-value <0.01 |
| **QOL Scales** |  |  |  |
| Diabetes-focused scale | 1.18 (0.41 to 1.95), 95.6% (n=14) | -* | -* |
| Generic scale | 2.12 (0.20 to 4.05), 97.3% (n=13) | - | - |
| Test for subgroup difference | Q=0.80, p-value=0.37 | - | - |
|  |  |  |  |

SMD: Standardized mean difference, QOL: Quality of life, PCS: Physical component summary, MCS: Mental component summary

*Subgroup analysis based on QoL scales for PCS and MCS was not possible. All studies involved used a generic QOL scale.

**Meta regression (duration)**

**Mean:** Q=1.13, p-value= 0.28

**PCS:** Q=0.36, p-value= 0.546

**MCS:** Q=-0.035, p-value= 0.35

**Meta-analysis of Mean QoL using different correlation coefficients (r)**

**r = 0.4:** SMD 1.44 (0.59 to 2.28), p-value=0.0008, I2=96%

**r = 0.8:** SMD 2.26 (1.01 to 3.50), p-value=0.0004, I2=97.3%
