# Supplementary material for: The effect of health behavior interventions to manage Type 2 diabetes on the quality of life in low-and middle-income countries: A systematic review and meta-analysis
Source: PLoS One. 2023 Oct 16;18(10):e0293028. doi: 10.1371/journal.pone.0293028 (PMC10578590; doi:10.1371/journal.pone.0293028)
Supplement: S7 Table — (DOCX) [file pone.0293028.s012.docx]

**S7 Table. List of studies excluded during full-text screening**

| Number of studies | Author | Reasons of exclusion |
| --- | --- | --- |
| 10 | Dadgostar 2016  Davis 2012  Guldbrand 2014  Izquierdo 2003  Kulzer 2007  Praet 2008  S 2018  Sukala 2013  Hortensius 2018  Wycherley 2014 | Absence of a control group |
| 24 | Baron 2017  Huang 2021  Groener 2015  Guo 2021  McCarron 2000  Ramanath 2011  Keeratiyutawong 2006  Sykes 2004  VanRooijen 2005 | Full text article not available |
| 52 | Alalwan 2020  Armitage 2021  Barham 2011  Barrett 2018  BohingamuMudiyanselage 2019  Cezaretto 2017  Chan 2018  Collins 2022  Duijzer 2019  Duijzer 2017  Dunbar 2015  Ebert 2017  Florez 2012  Freund 2016  Grey 2000  Griffiths 2005  Irvine 2011  Iversen 2020  Kan 2017  Kennedy 2013  Kuznetsov 2015  Leal 2017  Lear 2021  Li 2017  Liu 2013  Mager 2017  McDermott 2014  Nanditha 2020  Newby 2017  Oksman 2017  Pyatak 2018  Rhodes 2018  Rosas 2016  Schmitt 2022  Smith 2019  Tankova 2004  Tovote 2014  vanderWeegen 2015  vanSon 2014  vanWier 2013  Webb 2012  Weinger 2011  Adepu 2011  DeAzevedo 2017  Eriksson 2006  Keerthi 2017  Kerr 2002  VanDenDonk 2013  VanSon 2013  Bartlett 2011  Aira 2013  Yu 2020 | Study population were not T2DM, or were a mix of T2DM along with other people with other disease conditions |
| 46 | Abubakar 2021  Adams 2019  Barringer 2003  Cinar 2014  Corley 2018  Coventry 2015  Dalsgaard 2020  DomÃ­nguez-MuÃ±oz 2020  Ell 2011  Gepner 2015  Halperin 2014  Janssen 2009  Katalenich 2015  Kuo 2019  Logtenberg 2007  Lu 2011  MansanoPletsch 2021  MÃ©nard 2005  MÃ©nard 2007  Munsour 2020  Pitale 2005  Russell 2019  Segal 2016  Sharp 2018  Simonson 2018  Tajaddini 2021  vandenDonk 2010  Woo 2007  Xu 2018  Yalcin 2008  Che 2021  Ding 2015  Egede 2021  Gherman 2017  Katon 2010  Noviani 2020  Seyam 2020  Xu 2021  Bahadlr 2020  Ojieabu 2020  Ramanath 2012  Sriram 2011  Ramal 2018  Tu 2020  Frei 2014  Hajati 2021 | Not health behaviour intervention |
| 39 | Abdelbasset 2020  Agarwal 2019  Boaz 2009  Bosma 2011  Botton 2018  Bujnowska-Fedak 2011  D'EramoMelkus 2010  Gillett 2010  Griva 2019  Hay 2012  Heinrich 2010  Hendricks 2000  Hua 2022  Kirk 2001  LabrunÃ©e 2012  Mash 2015  Metz 2000  Rosales 2021  Simon 2021  Skelly 2009  Skoro-Kondza 2009  Steed 2005  Tang 2014  Trento 2010  Wolever 2017  Zaghloul 2020  Adepu 2007  Holmen 2014  Lindberg 2017  Marios 2012  Sazlina 2015  Iram 2010  Coyle 2012  Handley 2008  Beverly 2013  Simon 2008  Nam 2012  Bunner 2015  Forjuoh 2014 | Inadequate data on QOL outcome measure |
| 33 | Adams 2015  AhmadSharoni 2018  ArmaniKian 2018  Cinar 2014  doRosÃ¡rioPinto 2017  Doggrell 2002  Dutton 2015  Hsu 2021  Izgu 2020  Kopp 2012  Lincoln 2008  Long 2005  Malanda 2011  Murray 2017  Or 2016  Piatt 2011  Rozworska 2020  Schulte 2020  Skinner 2014  Smith 2000  Sugiyama 2015  Weymann 2015  Wu 2018  Yadav 2021  Yamada 2014  Yu 2019  Zheng 2019  Gehlawat 2019  Quinn 2017  Smith 2004  Kahleova 2011  Dixit 2014  Shah 2014 | QOL outcomes not reported |
| 12 | Albikawi 2016  Chen 2019  Ell 2010  Ritzwoller 2006  Shaya 2014  Tang 2010  Wang 2022  Williams 2014  BalchaHailu 2021  Garner 2021  WÃ¤ndell 2012  Thind 2019 | Not an RCT |
| 8 | Berk 2012  Chernyak 2010  Drewelow 2012  Mash 2012  Ng'ang'a 2020  Ramallo-FariÃ±a 2015  Seuring 2019  Zeitler 2007 | Study protocol |
| 2 | Watson 2018  Wayne 2015 | Wrong comparator |
| 2 | Delvarianzadeh 2006  Hassani 2018 | Not in English language |
| 1 | Taylor 2020 | Book report |
| 1 | Varming 2019 | Wrong indication |
| 156 |  | Studies conducted in high-income countries |
