# Supplementary material for: The effect of health behavior interventions to manage Type 2 diabetes on the quality of life in low-and middle-income countries: A systematic review and meta-analysis
Source: PLoS One. 2023 Oct 16;18(10):e0293028. doi: 10.1371/journal.pone.0293028 (PMC10578590; doi:10.1371/journal.pone.0293028)
Supplement: Attachment — Submitted filename: Response to Reviewers comments.docx [file pone.0293028.s015.docx]

The Editor-in-Chief
PLOS One

Subject: Submission of revised manuscript on “The effect of health behavior interventions to manage Type 2 diabetes on the quality of life in low-and middle-income countries: a systematic review and meta-analysis”

Thank you for providing us with the opportunity to submit a revised manuscript “The effect of health behavior interventions to manage Type 2 diabetes on the quality of life in low-and middle-income countries: a systematic review and meta-analysis” for consideration of publication in PLOS One.

We express our gratitude to the reviewers for providing feedback and their insightful comments on the manuscript, which certainly have helped to enhance the quality of the manuscript. We would like to confirm that the reference list is complete and correct and no retracted papers have been cited.

Please find a point-by-point response to the editor’s and reviewers’ comments below. Author’s responses are marked blue. All page and line numbers refer to the track changed version of the revised manuscript.

**Reviewer #1: The authors have addressed many of the suggestions from the previous peer review. I still believe that there can be more improvements to ensure current review methodology is followed and that the conclusions about the findings closely reflect the data.**

Thank you for your thoughtful comment. We have addressed all your comments.

Major comments:

1. I strongly object to using lack of feasibility of blinding as a reason to not rate the studies at risk of bias from lack of blinding. There is still the risk of bias regardless of blinding not being practical. This is commonly mentioned at training workshops to avoid doing. The authors may not think the risk is that serious to potentially rate down during GRADE, but the risk of bias from lack of blinding does exist.

Thank you for the comment. Upon further consideration and discussion with the co-authors, we agree with the reviewer and acknowledge that the lack of blinding poses a risk of bias in behavioral interventions regardless of whether blinding was feasible or not. Hence, studies not reporting blinding of either the participants, personnel or outcome assessors were given a score of “0” and marked as “N” for “No” under the respective items about blinding of participants, personnel or outcome assessors in the 13-item Joanna Briggs Institute (JBI) Critical Appraisal Checklist for Randomized Controlled Trials. We have detailed out all this information in the Quality Assessment section under Methods and results both, as following (please see pages 8-9, lines 160-180):

In Methods:

“The 13-item Joanna Briggs Institute (JBI) Critical Appraisal *Checklist for Randomized Controlled Trials was used to assess methodological quality of the selected studies [26]. Two reviewers (AK and PD) independently assessed the risk of bias. Any disagreements were discussed with the team members until consensus was reached.* *Each item in the JBI checklist was scored one if they fulfilled the criteria for that item and scored zero if they did not fulfil the criteria. For example, if a study reported blinding of outcome assessors, then the study was scored ‘1’ for the item about blinding of outcome assessors, whereas, if the study did not report blinding of outcome assessors, then the study was scored ‘0’. Summary scores were obtained for each selected studies by adding the item-specific scores. The quality of the studies was then rated as good (≥8), fair (6-7), or poor (≤5) based on the summary scores [27]. In addition, the quality of evidence across RCTs included in the meta-analysis was assessed by two reviewers (AK and PD) using the Grading of Recommendations, Assessment, Development and Evaluation (GRADE) approach, and rated as high, moderate, low or very low [28]. Since all included studies were RCTs, the rating began with a high-certainty rating. Then, the quality was upgraded or downgraded based on the following criteria: i) quality across the studies as determined by the JBI Critical Appraisal Checklist for Randomized Controlled Trials; ii) inconsistency/heterogeneity level; iii) indirectness of evidence; iv) imprecise results (wide 95% CI i.e., > 0.8 SMD); and v) publication bias (visual inspection of funnel plot) [29]. If the majority of the RCTs scored ≤ 5 on the JBI scale, the certainty of evidence was downgraded two places whereas, if the effect size was large (SMD ≥ 0.8), then the evidence was upgraded one place [29].”*

From the Results section, we have removed the statement “*Blinding of participants is generally difficult in behavioral interventions due to participants being aware whether they receive the intervention, hence studies that did not address or clarify blinding were not downgraded for those risks or uncertainties.”* (page 31, lines 283-286). We have also added a description in the Results section, which read as follows:

“*The overall quality of the studies ranged from “fair” to “good” after obtaining a summary score of at least six in the quality assessment. No study was rated as poor quality as none obtained a summary score of five or less.”* (please see page 31, lines 278-280)

2. The main text conclusions paragraph still mentions that the community setting is "likely" an impact on PCS and MCS findings, and this should be downplayed substantially.

We thank the reviewer for noting this. We have removed the statement about community setting having an impact on the PCS and MCS findings. The removed statement is in page 39 lines 480-482 of the track changed document.

3. Now that the authors have clarified that their results represent the immediate effects after the interventions, they should add the caveat that their findings are for the short-term QOL of participants and that that any lasting/sustained effect was not examined in this review. I think the term "follow-up effect" (not too sure if that is a used term) should be removed with focus that the authors chose to keep things consistent across studies using a similar timepoint.

Thank you for this comment. Since we measured the immediate post-intervention effects of the interventions and didn’t measure the longer-term effect of the interventions, we have added it as one of the limitations of the study in page 38-39 lines 466-469. The statement now reads as follows:

*“Thirdly, the findings of this meta-analysis only related to short-term QOL (i.e., immediate post-intervention effects) and any lasting/sustained effect was not examined in this review as there was no sufficient data to examine longer-term effects on QOL.”*

We have removed the term “follow-up effect” and revised the statement, which now reads as follows (page 10 lines 202-206):

*“For studies with QOL measurements at multiple time-points, only the measurements at baseline and following intervention completion (post-intervention) were included in the meta-analysis to maintain consistency across studies using a similar timepoint and improve homogeneity in data extraction.”*

Minor:

abstract: suggest to remove "significant" in the sentence about the mean QOL findings.

Thank you for the comment. The word “significant” has been removed from the statement, which now reads as below (please see page 2, line 34):

*“There was moderate quality evidence from the meta-analysis of mean QOL (n=25) that health behavior intervention improved the QOL of people with T2DM (SMD=1.62, 95%CI=0.65-2.60 I^2^=0.96, p=0.001).”*

methods: delete the first sentence in the data extraction section (this refers to eligibility criteria); was <=5 or <=6 used for GRADE study limitations domain? This seems inconsistent with the risk of bias categories; please mention that the added subgroups for study quality and measurement tool were added post hoc (after protocol).

Thank you for the comment. The first sentence from the data extraction section in the manuscript body, which initially read as *“Data was extracted only from the RCTs conducted in LMICs”* has been removed. (see page 8, line 153)

≤5 was used as the GRADE study limitation domain to be consistent with the risk of bias categories. We have corrected the inconsistencies and replaced ≤6 with ≤5. The sentence now reads as below *(*please see page 9, lines 177-180*)*:

*“If the majority of the RCTs scored ≤5 on the JBI scale, the certainty of evidence was downgraded two places whereas, if the effect size was large (SMD ≥ 0.8), then the evidence was upgraded one place [29].”*

We have added a statement about performing post-hoc subgroup analyses based on study quality and QOL scale, which reads as follows:

*“Sub-group analyses based on QOL scale and methodology quality were added post hoc.” (see page 10 lines 213-214)*

results: are the findings for the overall QOL analysis SMD 1.46 or 1.62? this has changed since the original submission and is not consistent throughout the paper.

Thank you for the comment. The correct SMD is 1.62. We have made necessary correction at page 32-33 line 323-324). The corrected sentence now reads as follows:

“*However, due to large effect size (SMD: 1.62), the studies were upgraded by one level.”*

I had hoped that the authors would have removed the "influencer" and outlier analyses, which are not current practice due to their focusing on results rather than clinical and methodological differences between studies, and would encourage them to reconsider this. The fact that these analyses did not impact the findings is not a good reason to keep them in the paper. We do not just remove/exclude a study because of it's findings being "different" from others, unless there is a methodological or clinical reason to do so. What if they study was actually the best conducted and largest??

Thank you for the comment. We have removed the “influence” and “outlier” analyses as suggested by the reviewer. We have removed all the following texts in relation to influence and outlier analyses from the manuscript:

*“Outliers were identified statistically and graphically.” (page 10, line 199)*

*“Sensitivity analyses were performed using a leave-one-out method, where individual trials are excluded one at a time and the changes in overall results and heterogeneity are explored.” (page 10, lines 207-208)*

*“Influence analyses (S2 Fig) suggested that the overall meta-analysis was sensitive to the study by Safavi et al (74). Excluding this study did not change the direction of the effect (SMD: 1.17, 95% CI: 0.70 to 1.64, I^2^: 0.95).” (page 32, lines 303-305)*

*“Outlier analysis identified nine studies as outliers [8, 46, 48, 50, 52, 55, 61, 63, 74]. Excluding these studies reduced the heterogeneity slightly (to 90.1%) and the magnitude but not the overall direction of the effect (SMD: 1.14, 95% CI: 0.83 to 1.45, p-value<0.001).” (page 33, lines 326-328)*

*“Influence analyses (S4 Fig) suggested that the overall meta-analysis was sensitive to the study by Rias et al. [49]. Excluding this study did not change the direction or the significance of the effect (SMD: 0.458, 95% CI: -0.125 to 1.040, I2: 0.922).” (page 33, lines 336-338)*

*“Outlier analysis identified two studies as outliers [44, 49]. Excluding these studies reduced the heterogeneity to 0% and resulted in an overall significant effect (SMD 0.18, 95% CI: 0.03 to 0.34, p-value<0.05).” (page 34, lines 352-354)*

*“Influence analyses (S6 Fig) suggested that the overall meta-analysis was sensitive to the study by Sekhar et al. [44]. However, excluding this study did not change the direction or the significance of the effect (SMD: 0.094, 95% CI: -0.349 to 0.537, I2: 0.883).” (page 34, lines 361-363)*

*“Two studies were identified as outliers in the outlier analysis [44, 73]. Excluding these studies reduced the heterogeneity to 72.4% but not the overall direction of the effect (SMD 0.24, 95% CI: -0.17 to 0.65, p-value<0.256).” (page 35, lines 377-379)*

We have also removed the supporting information caption of sensitivity analyses, which initially read as below:

*“S2 Fig. Sensitivity analysis of Mean Quality of life” (page 47 line 790)*

*“S4 Fig. Sensitivity analysis of Mean Physical Component Summary” (page 47 line 792)*

*“S6 Fig. Sensitivity analysis of mean Mental Component Summary” (page 47 line 795)*

Lastly, I think the authors could consider focusing on the (moderate certainty) overall QOL findings and speak to these a bit more strongly as indicating benefit at least over the short term. One recommendation may be for more longer term follow-up to see if the effects are sustainable.

Thank you for this comment. We have rephrased the main text conclusion focusing on the moderate certainty of evidence of overall QOL findings indicating benefits over at least short period of time. The revised conclusion reads as follows (please see page 39-40 lines 478-493):

*“In conclusion, this systematic review and meta-analysis identified health behavior interventions targeted at improving T2DM management as effective strategies in improving the QOL of people with T2DM. The study demonstrated a moderate certainty of evidence for the overall QOL findings, indicating that the immediate post-intervention improvement on the overall QOL of people with T2DM is likely to be close to the true effect of the interventions on QOL over the short term. However, the low quality of evidence of PCS and MCS findings limits the strength of our conclusion from the pooled evidence. Due to the variability of the scales used in QOL measurement, the interpretation of our findings warrants caution. Studies with specific standardized scales are recommended for more robust estimates. Further research is needed to examine the long-term effectiveness of health behavior interventions on QOL. Furthermore, this review highlights the need for more well-designed RCTs of high methodological quality focusing on QOL as a primary outcome measure. The evidence generated from this review may guide future research and clinical practice and may derive policy implications for the management of T2DM in LMICs.”*

We have added a recommendation about the need for conducting further studies assessing the longer-term effects of the interventions; the statement reads as follows (see page 39 lines 489-490):

*“Further research is needed to examine the long-term effectiveness of health behaviour interventions on QOL.”*

**Reviewer #2: Thank you authors for addressing the comments raised. Congratulations on the job well done and this has improved the quality of the manuscript.**

Thank you. We appreciate the valuable feedback from the reviewers.
