# Supplementary material for: The effect of health behavior interventions to manage Type 2 diabetes on the quality of life in low-and middle-income countries: A systematic review and meta-analysis
Source: PLoS One. 2023 Oct 16;18(10):e0293028. doi: 10.1371/journal.pone.0293028 (PMC10578590; doi:10.1371/journal.pone.0293028)
Supplement: Attachment — Submitted filename: Reviewer comments.pdf [file pone.0293028.s013.pdf]

The effect of health behaviour interventions to manage Type 2 diabetes on the quality of life in low-and middle-income countries: a systematic review and meta-analysis

Abstract

Background

Limited research has assessed this effect in low and middle-income countries (LMICs), nor has it been systematically reviewed. Remember, the research on this area is available but the synthesis of the research done to this effect is limited.

Methods

- Was there a timeline for this review?
- Which methodology did you follow? Is it the JBI systematic review methodology?

Conclusion

- What recommendation can be given based on your study results?

Ker words

- Rather say health behaviour intervention
- Randomised control trial is not a key word, kindly remove it

Introduction

However, despite QOL being an important measure in T2DM management, studies assessing the effect of these interventions on the QOL of people with T2DM in LMICs are limited. Remember, this is not primary research. You are looking at synthesis of the available studies whether they are few or not. Therefore, your motivation for the review should not be based on this.

Methodology

Data sources and searches

- This systematic review adhered to the Preferred Reporting Items for Systematic Reviews and Meta-Analysis (PRISMA) guideline [20]. The review protocol was registered in the PROSPERO International Prospective Register of systematic reviews (Registration ID 90 CRD42022323184). Give a subheading to this section as **Reporting and registration of the review.** Also give a date when the protocol was registered.
- Kindly add the date for the data searches, how long did it last?
- Online databases PUBMED/MEDLINE, SCOPUS, CINAHL, Embase, Web of Science and PsycINFO were searched using the pre-defined search terms. How did you come out with the predefined search terms? Did you use PICO/PIO?
- A manual search of reference lists of included studies was also conducted to identify additional studies that met the inclusion criteria. What determined your inclusion and exclusion criteria? This needs to be covered maybe separately so that it becomes clearer to the readers.

- Then data was extracted only from the RCTs conducted in LMICs. Why are you talking of data extraction at this stage of searching in data bases? Whatever you search in data bases is taken to a systematic review app (either covidence or Ryann) before you even start of selection and extraction.
- How did the authors define the LMIC? How many countries make up the LMIC?

Study selection

- Only studies with an RCT design were included in this review to ensure that the evidence 103 provided on the effectiveness of interventions was robust. When multiple articles from the 104 same study/studies were found, only the article that reported the most relevant QOL 105 information/outcome was included. All types of health behavior interventions were included 106 regardless of the setting. Only studies conducted between January 2000 and May 2022 and published in English language were included in the review, as examining QOL in individuals with T2DM is a relatively new concept. Studies were included if they 1) examined any behavioral or educational interventions targeted at improving T2DM management among people with new or established diagnosis of T2DM; 2) reported QOL outcomes using validated 111 QOL measures, both pre- and post-intervention, as a primary or secondary outcome; 3) were conducted within an RCT design; and 4) were conducted in LMICs [21]. Studies were excluded if they 1) were published in languages other than English; 2) had therapeutic or pharmacological intervention strategies; 3) had mixed study population of Type 1 and 2 diabetes with no separate data reported for T2DM population; 4) were observational studies or reported in reviews, editorials, theses, books, short communication; or 5) presented inadequate or unclear QOL data. This is the inclusion and exclusion criteria which should be presented separately.
- Selection of articles should start after all the selected articles from the data bases are in Covidence. Once in Covidence, you start with duplicate removal. After duplicate removal, outline the selection based on abstract and title first, then full text screening.
- Full texts of retrieved studies were then screened by the first reviewer (AK). Why the full text screening done by one reviewer? Because two reviewers are supposed to do independently with blind on, then solve the disagreements later.
- Any disagreements were resolved through consultation with other team members. What type of disagreements were resolved or were there any?
- Add the selection dates, how long did the selection take?

Data Extraction

- Kindly add the dates for the data extraction
